# Glycoprotein Ibα‐Dependent Platelet Activation is Essential for Tumor Cell–Platelet Interaction and Experimental Metastasis

**DOI:** 10.1002/mco2.70217

**Published:** 2025-06-09

**Authors:** Kangxi Zhou, Qing Li, Yue Xia, Chenglin Sun, Jing Wang, Yueyue Sun, Xinxin Ge, Mengnan Yang, Yu Li, Sai Zhang, Lili Zhao, Chunliang Liu, Khan Muhammad Shoaib, Weiling Xiao, Renping Hu, Kesheng Dai, Rong Yan

**Affiliations:** ^1^ Jiangsu Institute of Hematology The First Affiliated Hospital of Soochow University, Cyrus Tang Medical Institute Suzhou Medical College, Soochow University, NHC Key Laboratory of Thrombosis and Hemostasis, National Clinical Research Center for Hematological Diseases Suzhou Jiangsu China

**Keywords:** glycoprotein Ibα, metastasis, platelet activation, tumor cell–platelet interaction

## Abstract

Metastasis is the main cause of cancer‐related deaths and the biggest challenge in improving cancer prognosis. Platelet–tumor cell aggregates are a prerequisite for hematogenous metastasis. However, the internal relation and molecular mechanism of platelets and their receptor glycoprotein (GP) Ibα in platelet–tumor cell interaction and metastasis remain elusive. Here, we find that in the absence of the full‐length GPIbα or its cytoplasmic tail, platelets maintain a more resting state and exhibit reduced tumor cell‐induced platelet activation. The deficiency of the cytoplasmic tail of GPIbα inhibits tumor cell–platelet interaction, platelet‐induced tumor cell migration and invasion, and metastasis. Using a state‐of‐the‐art spinning disk intravital microscopy, we observe a rapid accumulation of platelets on tumor cells, forming numerous tumor cell–platelet aggregates in vivo. We also find that the cytoplasmic tail of GPIbα regulates the tumor cell‐induced platelet protein kinase C‐α (PKCα) activation, and both the pharmacological inhibition and genetic ablation of platelet PKCα attenuate tumor cell‐induced platelet activation, tumor cell–platelet interaction, tumor cell migration and invasion, and metastasis. Overall, our findings reveal for the first time that GPIbα promotes experimental metastasis through its cytoplasmic tail‐regulated platelet activation, and suggest a potential target to regulate tumor hematogenous metastasis.

## 1 Introduction

Platelets, apart from their crucial role in thrombosis and hemostasis, have been increasingly implicated in sustaining tumor metastasis [1, 2]. Tumor cell metastasis is an important factor leading to 90% of cancer‐associated deaths [3, 4]. The relevance of platelets to hematogenous tumor metastasis has been corroborated [5, 6, 7]. When the tumor cells detach from the primary tumor and access the blood in the initial minutes, platelets are the first host cells they encounter [8]. Tumor cell‐activated platelets encase circulating tumor cells in a thrombus through adhesion molecules (such as P‐selectin, glycoprotein [GP] IIb/IIIa, etc.) to protect them from cytolysis by natural killer (NK) cells [9, 10]. Increased platelet activity promotes tumor metastasis and paraneoplastic thrombocytosis is predictive of poor prognosis in different types of solid tumors, such as ovarian cancer, colorectal cancer, and liver cancer [11, 12, 13, 14, 15]. Therefore, platelets are described as “first responders” and considered active participants in metastatic processes [16, 17].

More than half a century ago, platelet reduction or platelet function inhibition was found to be associated with antimetastatic effects [5, 7]. Recently, Shirai et al. [18] also demonstrated that a lowered but hemostatic platelet count reduced the risk of pulmonary metastasis. Particularly, clinical trials demonstrated a clear benefit of aspirin, which inhibits platelet function, in reducing mortality of solid tumors [19, 20]. However, despite speculations, the molecular mechanisms underlying the role of platelets in tumor metastasis have not been fully elucidated.

Glycoprotein Ibα (GPIbα) is a specific ligand‐binding subunit of the GPIb–IX complex on platelets [21, 22]. The GPIbα extracellular domain interacts with the von Willebrand factor (VWF), initiating platelet adhesion, hemostasis, and thrombus formation, which can lead to serious conditions, including myocardial infarction and stroke [23]. The GPIbα cytoplasmic domain contains binding sites for filamin A, which attaches the GPIb–IX complex to the cytoskeleton [24, 25], and 14‐3‐3ζ, an essential mediator of VWF–GPIbα‐mediated signaling [26, 27]. Several reports suggest that platelet GPIbα is associated with tumor malignancy [15, 28, 29]. Previous research found that the deficiency of GPIbα and its extracellular domain reduced the number of metastatic foci in the lung, suggesting that platelet GPIbα is the structurally relevant component of the GPIb–IX complex that contributes to metastasis [29]. Additionally, genetic dysfunction of GPIbα as well as anti‐GPIbα antibody treatment reduces nonalcoholic steatohepatitis and subsequent hepatocellular carcinoma development [15]. The variable number of tandem repeats polymorphism of the GPIbα gene is associated with increased risk for oral cancer [28]. Of note, for tumor growth, GPIbα has also been utilized as a marker for epithelial‐mesenchymal transition and tumor progression [30, 31].

Interestingly, a series of anti‐GPIbα antibodies, which target the GPIbα extracellular domain and inhibit VWF binding to GPIbα, reduced both experimental and spontaneous pulmonary metastasis of tumor cells [32]. However, another group reported that the Fab fragment of another anti‐GPIbα antibody, which also inhibits VWF binding to GPIbα, promoted metastasis [33]. Thus, the role of GPIbα in hematogenous metastasis and the underlying molecular mechanisms remain elusive.

In the present study, we found that GPIbα is essential for tumor cell‐induced platelet activation and tumor cell–platelet interaction, which assists the platelet “cloak” in encasing the tumor cells in a thrombus and safeguards tumor cells from immune surveillance to promote metastasis. Deficiency of the GPIbα cytoplasmic tail greatly decreases tumor cell‐induced platelet activation by regulating PKC activation, and the inhibition of PKCα‐dependent platelet activation by pharmacological inhibitors or genetic ablation of PKCα attenuates hematogenous metastasis.

## 2 Results

### 2.1 Gp1ba Deficiency Inhibits Tumor Cell‐Induced Platelet Activation and Experimental Metastasis

**FIGURE 1:**
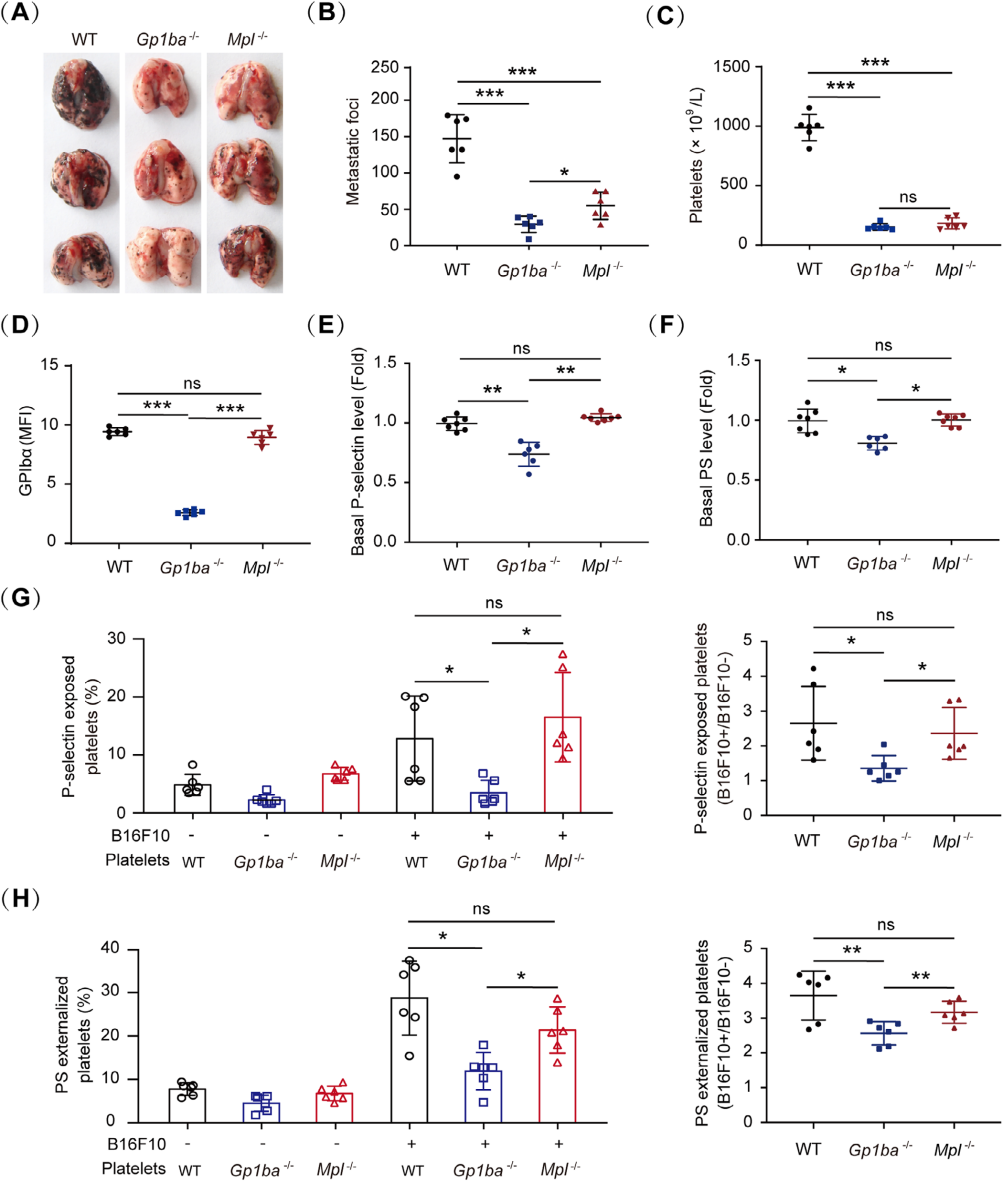
The role of GPIbα in metastasis. (A and B) Representative images (A) and the number of metastatic foci (B) in the lung of WT, *Gp1ba^−/−^* and *Mpl^−/−^* mice (*n* = 6 mice per group). (C) Platelet counts in WT, *Gp1ba^−/−^* and *Mpl^−/−^* mice (*n* = 6 mice per genotype). (D) Surface levels of GPIbα on wild‐type (WT), *Gp1ba^−/−^*, and *Mpl^−/−^* platelets analyzed by flow cytometry (*n* = 6 mice per genotype). MFI: mean fluorescence intensity. (E and F) Quantification plots of basal P‐selectin (E) and PS (F) levels on resting WT, *Gp1ba^−/−^*, and *Mpl^−/−^* platelets in PRP (WT and *Mpl^−/−^*, *n* = 7 mice; *Gp1ba^−/−^*, *n* = 6 mice). PS, phosphatidylserine. (G and H) Quantification plots of B16F10 cells‐induced P‐selectin (G) and PS (H) exposure on WT, *Gp1ba^−/−^*, and *Mpl^−/−^* platelets (*n* = 6 mice per group). One‐way ANOVA followed by Tukey's post hoc test in (B–H). Data are shown as mean ± SD (B–H). *p < 0.05, **p < 0.01, and ***p < 0.001. ns, not significant.

To investigate the role of GPIbα in tumor metastasis, an experimental pulmonary metastasis model was selected. Consistent with the literature [29], the number of pulmonary metastatic foci was significantly lower in GPIbα‐deficient (*Gp1ba* ^−/−^) mice compared with that in wild‐type (WT) mice 14 days after injection of B16F10 cells (Figure 1A,B). As it is known, low platelet counts attenuate metastasis [17]. Since *Gp1ba^−/−^* mice displayed severe macrothrombocytopenia (Figure 1C) [34], to distinguish the role of GPIbα in metastasis, *Mpl* ^−/−^ mice, which have low platelet counts but normal GPIbα expression in the platelets, were selected (Figure 1C,D). Expectedly, the number of pulmonary metastatic foci (Figure 1A,B) was significantly lower in *Mpl^−/−^* mice than in WT mice, which further demonstrates the crucial role of platelet count in metastasis. However, although *Gp1ba^−/−^* and *Mpl* ^−/−^ mice have almost the same platelet counts (platelet counts were “not statistically different” with the SD and *p* value) (Figure 1C), the number of pulmonary metastatic foci was obviously lower in *Gp1ba^−/−^* mice than that in *Mpl^−/−^* mice (Figure 1A,B), suggesting that other factors may regulate experimental pulmonary metastasis in *Gp1ba^−/−^* mice besides low platelet count.

Upon entering the blood vessel, tumor cells activate platelets to form platelet–cell aggregates, which protect tumor cells from deleterious shear stress [35] and the assault of NK cells [36] to fulfill distant dissemination. To investigate whether GPIbα deficiency‐attenuated tumor metastasis is related to decreased platelet activation, basal levels and tumor cell‐induced surface exposure of P‐selectin and phosphatidylserine (PS), which are two markers for platelet activation and have been shown to mediate the tumor cell–platelet interaction [37, 38, 39], were compared among WT, *Gp1ba^−/−^*, and *Mpl^−/−^* platelets. Interestingly, compared with the WT and *Mpl* ^−/−^ platelets, the resting *Gp1ba^−/−^* platelets displayed lower basal levels of P‐selectin (Figures 1E and S1A) and PS (Figures 1F and S1B) on the platelet surface. Moreover, B16F10 cells incurred less P‐selectin (Figures 1G and S1C) and PS exposure (Figures 1H and S1D) on the *Gp1ba^−/−^* platelets. The total protein and mRNA levels of P‐selectin were not affected in *Gp1ba^−/−^* platelets (Figure S2). These data suggest that *Gp1ba^−/−^* platelets are in more resting states and that GPIbα deficiency inhibits tumor cell‐induced platelet activation and pulmonary metastasis.

### 2.2 The GPIbα Cytoplasmic Tail is Essential for Tumor Cell‐Induced Platelet Activation

**FIGURE 2:**
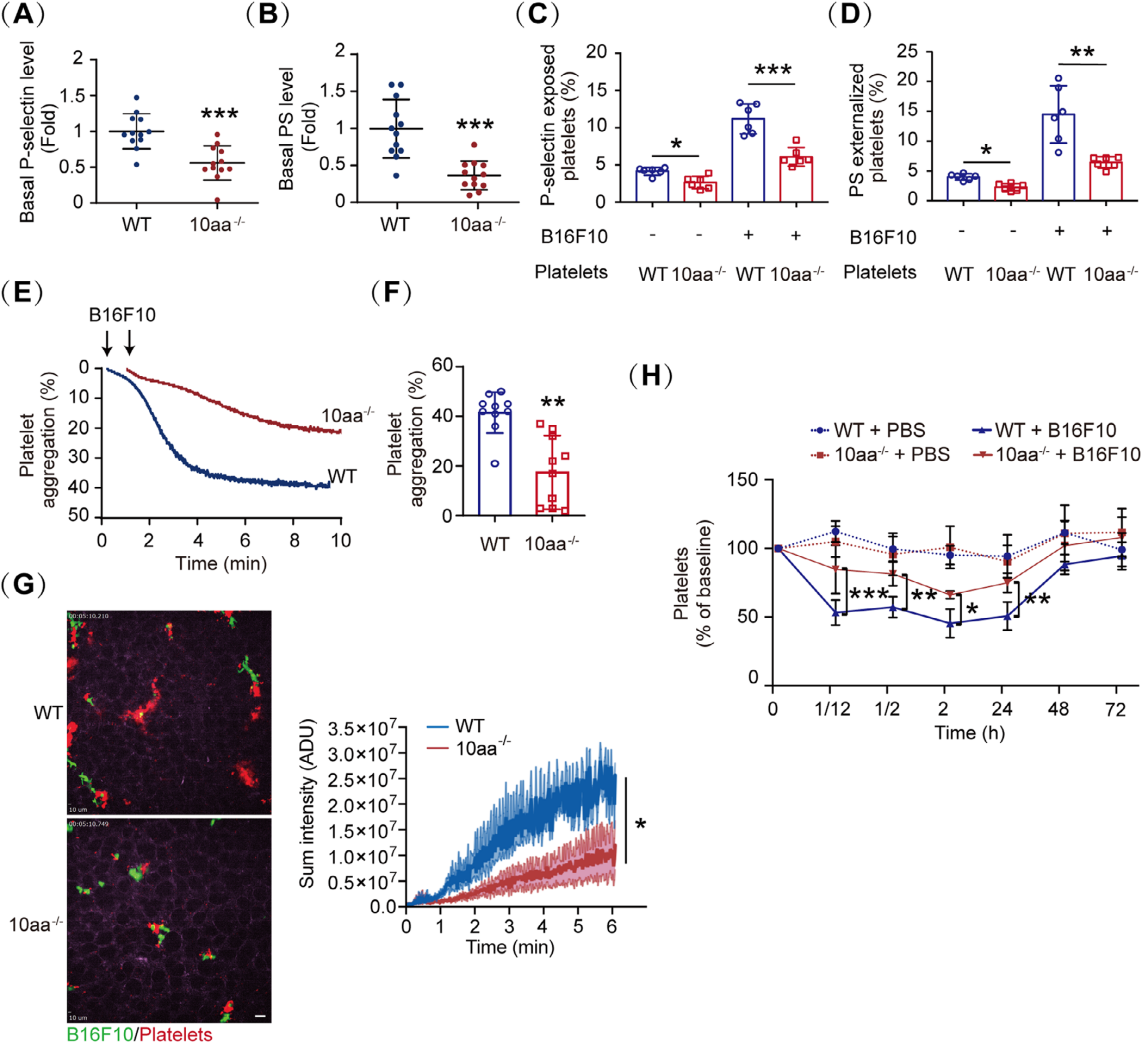
The GPIbα cytoplasmic tail is essential for tumor cell‐induced platelet activation. (A and B) Quantification plots of basal P‐selectin (A) and PS (B) levels on resting WT and 10aa^−/−^ platelets in PRP (*n* = 12 mice per genotype). (C and D) Quantification plots of B16F10 cells‐induced P‐selectin (C) and PS (D) exposure on WT and *10aa* ^−/−^ platelets (*n* = 6 mice per group). (E and F) Representative traces of B16F10 cell‐induced WT and 10aa^−/−^ platelet aggregation (E) and the quantification of maximal aggregation rate (F) (*n* = 10 independent experiments). (G) Representative state‐of‐the‐art spinning disk intravital microscopy (SDIM) images (left) and quantification (right) of tumor cell‐induced thrombus fluorescence (red) of live WT and 10aa^−/−^ lung 5 min after intravenous injection of B16F10 cells. ADU: analog‐to‐digital unit. Scale bar: 30 µm. (H) B16F10 cells induced platelet clearance in WT and 10aa^−/−^ mice. Two‐tailed Student's *t*‐test in (A), (B), and (F). One‐way ANOVA followed by Tukey's post hoc test in (C) and (D). Mann–Whitney *U*‐test in panel (G). Two‐way ANOVA followed by Dunnett's post hoc test in (H). Data are shown as mean ± SD (A–D and F–H). *p < 0.05, **p < 0.01, and ***p < 0.001.

GPIbα cytoplasmic tail contains 14‐3‐3ζ binding site, which is important for VWF–GPIbα‐mediated signaling [23, 24, 25]. We recently found that the GPIbα cytoplasmic tail also regulates platelet general activation beyond the VWF–GPIbα axis [40]. Thus, we next used a previously generated mouse model with GPIbα C‐terminal residue‐deleted (10aa^−/−^) [40] to study the role of this region in tumor cell‐induced platelet activation. Similar to *Gp1ba^−/−^* platelets, 10aa^−/−^ platelets exhibited not only lower basal P‐selectin (Figures 2A and S3A) and PS (Figures 2B and S3B) levels but also lower tumor cell‐induced P‐selectin (Figures 2C and S3C) and PS exposure (Figures 2D and S3D). Moreover, tumor cell‐induced platelet aggregation was significantly reduced in 10aa^−/−^ platelets (Figure 2E,F). Additionally, using a state‐of‐the‐art spinning disk intravital microscopy (SDIM) allowed us to delineate the interaction of tumor cells with platelets in the murine lung. After intravenous injection of B16F10 cells, we observed a rapid accumulation of platelets on almost all tumor cells, forming numerous tumor cell–platelet aggregates (or tumor cell‐induced microthrombus) in the initial minutes in the lung of WT mice (Figure 2G and Video S1), resulting in a reduction of circulation platelets (Figure 2H). However, only a few tumor cell–platelet aggregates (or tumor cell‐induced microthrombus) formed (Figure 2G and Video S2) in the 10aa^−/−^ murine lung, and there was only a small decrease in platelet in 10aa^−/−^ mice (Figure 2H). Collectively, these results demonstrate that the cytoplasmic tail of GPIbα is essential for tumor cell‐induced platelet activation.

### 2.3 The GPIbα Cytoplasmic Tail Deficiency Inhibits B16F10 Cell–Platelet Interaction and Experimental Metastasis

**FIGURE 3:**
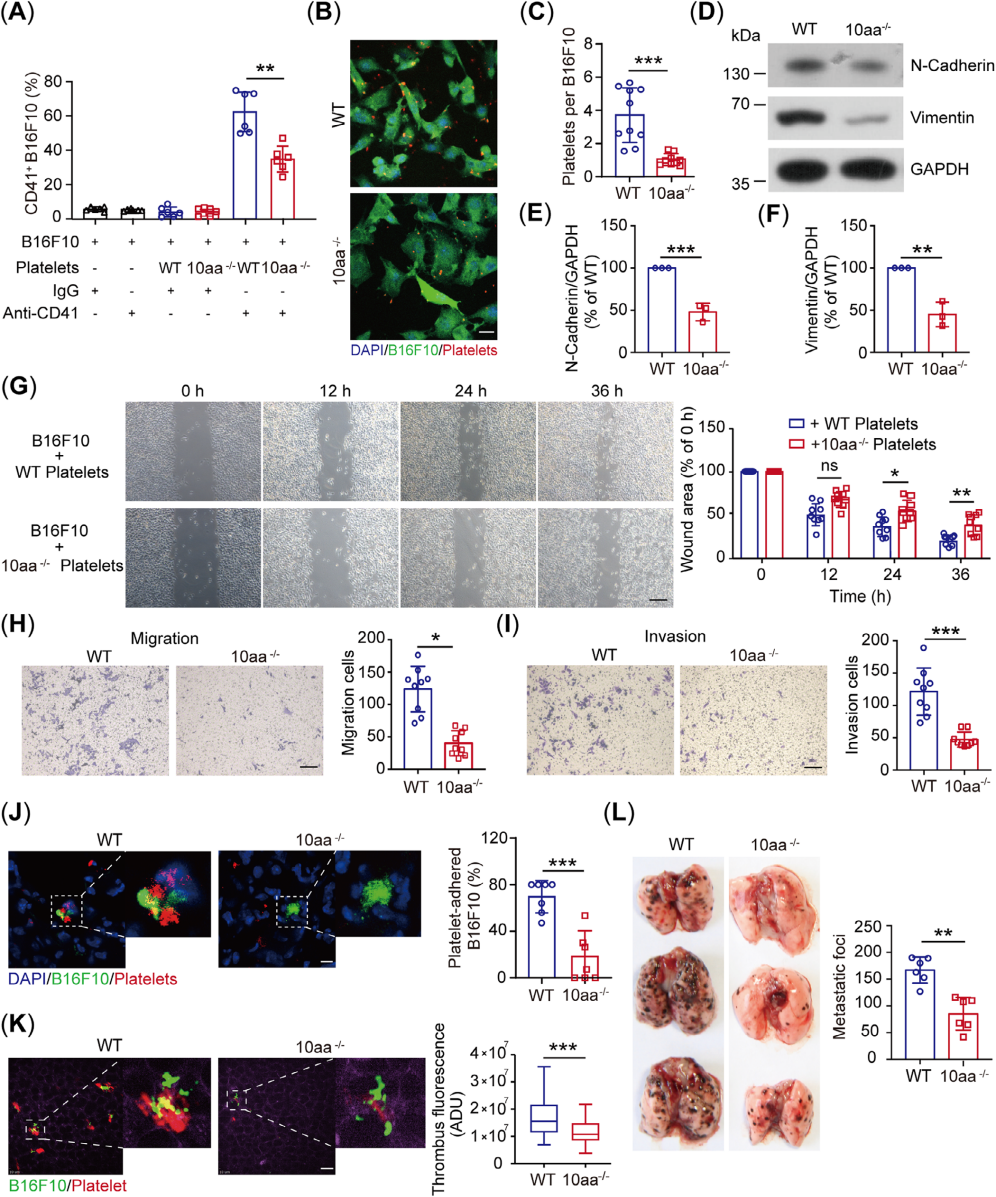
The GPIbα cytoplasmic tail deficiency inhibits the interaction of B16F10 cells with platelets and experimental metastasis. (A) The in vitro adhesion of WT and 10aa^−/−^ platelets to B16F10 cells was analyzed by flow cytometry using an antiplatelet CD41 antibody (*n* = 6 independent experiments). (B and C) Representative confocal images (B) and quantification (C) of the interaction of GFP–B16F10 with WT and 10aa^−/−^ platelets in vitro (*n* = 10 visual fields from 10 mice of each genotype). Scale bar: 25 µm. D‐F, Representative blot (D) and quantification of N‐cadherin (E) and vimentin (F) in B16F10 cells treated with WT and 10aa^−/−^ platelets (*n* = 3 mice per genotype). (G) Representative images of B16F10 cell wound‐healing (left) and quantification plots (right) of the wound areas of B16F10 cells incubated with washed WT and 10aa^−/−^ platelets (*n* = 9 visual fields from three mice of each genotype). Scale bar: 30 µm. (H and I) Representative images (left) and quantification plots (right) of invasion (H) and migration (I) of B16F10 cells incubated with WT and 10aa^−/−^ platelets (*n* = 9 visual fields from three mice of each genotype). Scale bar: 30 µm. (J) Representative confocal images (left) and quantification (right) of the interaction of GFP–B16F10 with WT and 10aa^−/−^ platelets in vivo (*n* = 7 visual fields from seven mice of each genotype). Scale bar: 25 µm. (K) Representative SDIM images (left) and quantification (right) of tumor cell‐induced thrombus fluorescence or B16F10 cell area fluorescence in vivo of WT and 10aa^−/−^ 6 h after the intravenous injection of B16F10 cells (*n* = 60 visual fields from five mice of each genotype). ADU: analog‐to‐digital unit. Scale bar: 30 µm. L, Representative images (left) and the number of metastatic foci (right) in the lung of WT and 10aa^−/−^ mice (*n* = 6 mice per group). One‐way ANOVA followed by Tukey's post hoc test in (A). Two‐tailed Student's *t*‐test in (C), (E), (F), and (H)–(L). Two‐way ANOVA followed by Bonferroni's post hoc test in (G). Data are shown as mean ± SD (A, C, E–J, and L) and min to max (K). *p < 0.05, **p < 0.01, and ***p < 0.001. ns, not significant.

Data have indicated that activated platelets wrap around the tumor cells to protect them from immune surveillance [9, 41]. Therefore, we investigated the interaction of B16F10 with platelets in vitro and found that fewer 10aa^−/−^ platelets interacted with B16F10 cells than WT platelets (Figures 3A–C and S4). The interaction of platelets with B16F10 cells promoted B16F10 cell migration and invasion in vitro [42, 43]. Then, we examined the metastatic tendency between WT and 10aa^−/−^ platelet‐treated B16F10 cells and found that the expression levels of mesenchymal markers, including N‐cadherin and vimentin, were decreased in 10aa^−/−^ platelet‐treated cells compared with the WT platelet‐treated cells (Figure 3D–F). Furthermore, wound‐healing and transwell assays showed that the B16F10 cells treated with 10aa^−/−^ platelets exhibited lower migratory activity than the WT platelet‐treated cells (Figure 3G–I). The key to successful metastasis is whether platelets can encase circulating tumor cells to protect them from cytolysis by NK cells in the initial hours after tumor cells enter into circulation [44]. Thus, we examined the tumor cell–platelet interaction in the lung 6 h after GFP–B16F10 cell injection both by confocal microscopy and SDIM. Fewer platelets interacted with B16F10 cells were observed in the cryosection of 10aa^−/−^ murine lung by confocal microscopy (Figure 3J), and there were fewer tumor cell–platelet aggregates in the live 10aa^−/−^ murine lung (Figure 3K and Videos S3 and S4). Furthermore, the number of metastatic nodules in the lung was significantly reduced in 10aa^−/−^ mice (Figure 3L). Altogether, our data indicate that the GPIbα cytoplasmic tail deficiency inhibits the interaction of B16F10 cells with platelets and attenuates the metastatic tendency of platelet‐treated B16F10 cells, resulting in a decrease in the number of metastatic lung nodules.

### 2.4 The GPIbα Cytoplasmic Tail‐Regulated PKCα Activation is Important for Tumor Cell‐Induced Platelet Activation, Interaction, and Tumor Cell Metastasis

**FIGURE 4:**
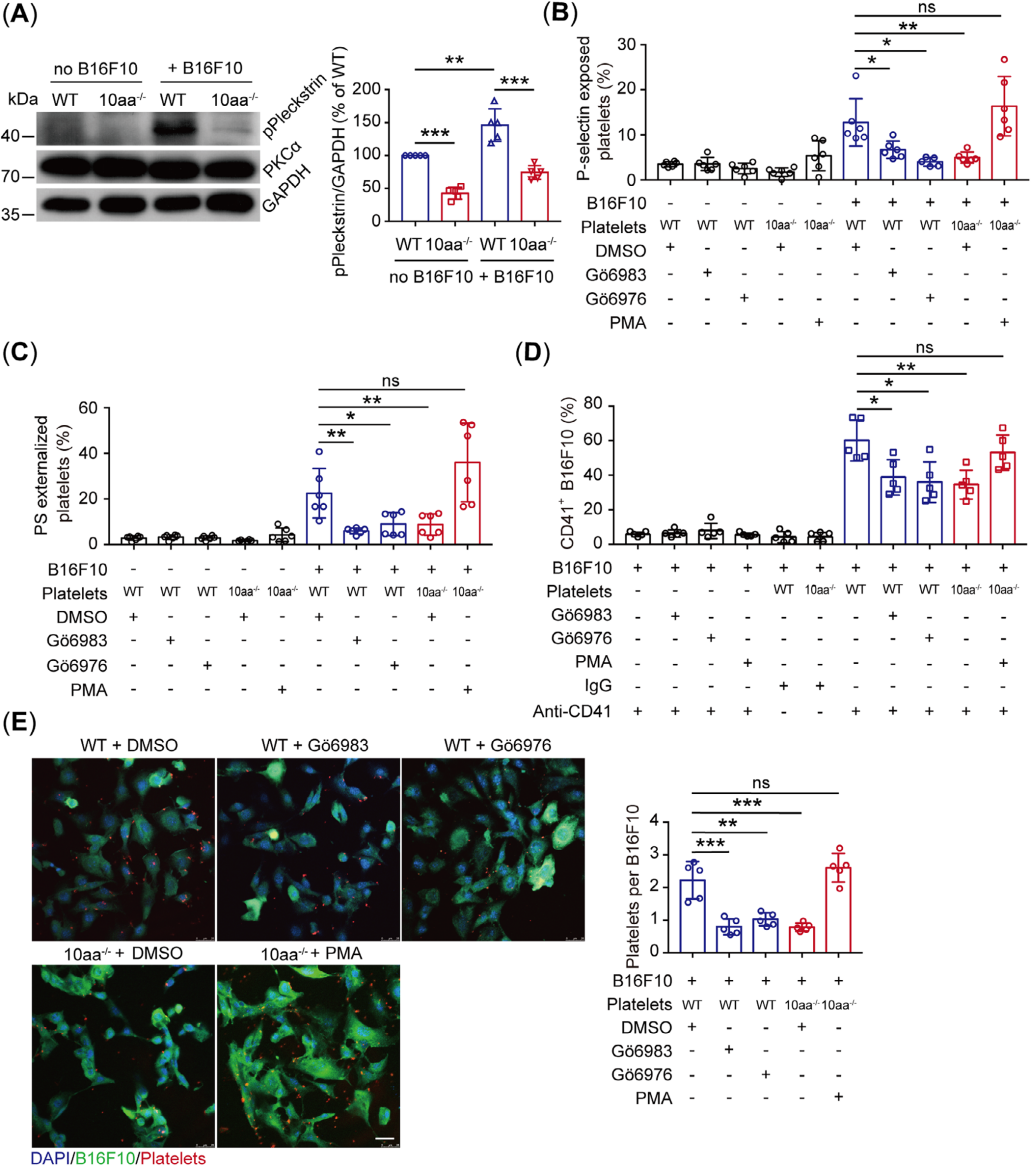
The GPIbα cytoplasmic tail supports tumor cell‐induced platelet activation and interaction by regulating PKC activity. (A) Representative blot of PKC substrate phosphorylation in resting WT and 10aa^‐/‐^ platelets and B16F10‐treated WT and 10aa^‐/‐^ platelets, and quantification of densitometry in the blots (*n* = 5 mice per genotype). (B and C) B16F10 cell‐induced P‐selectin (B) and PS (C) exposure on washed WT and 10aa^‐/‐^ platelets (*n* = 6 independent experiments). (D) The in vitro adhesion of WT and 10aa^‐/‐^ platelets to B16F10 cells was analyzed by flow cytometry using an antiplatelet CD41 antibody (*n* = 5 independent experiments). (E) Representative confocal images and quantification of the interaction of GFP–B16F10 with WT and 10aa^‐/‐^ platelets in vitro (*n* = 5 visual fields from five mice of each genotype). Scale bar: 25 µm. One‐way ANOVA followed by Tukey's post hoc test in (A–E). Data are shown as mean ± SD (A–E). *p < 0.05, **p < 0.01, and ***p < 0.001. ns, not significant.

**FIGURE 5:**
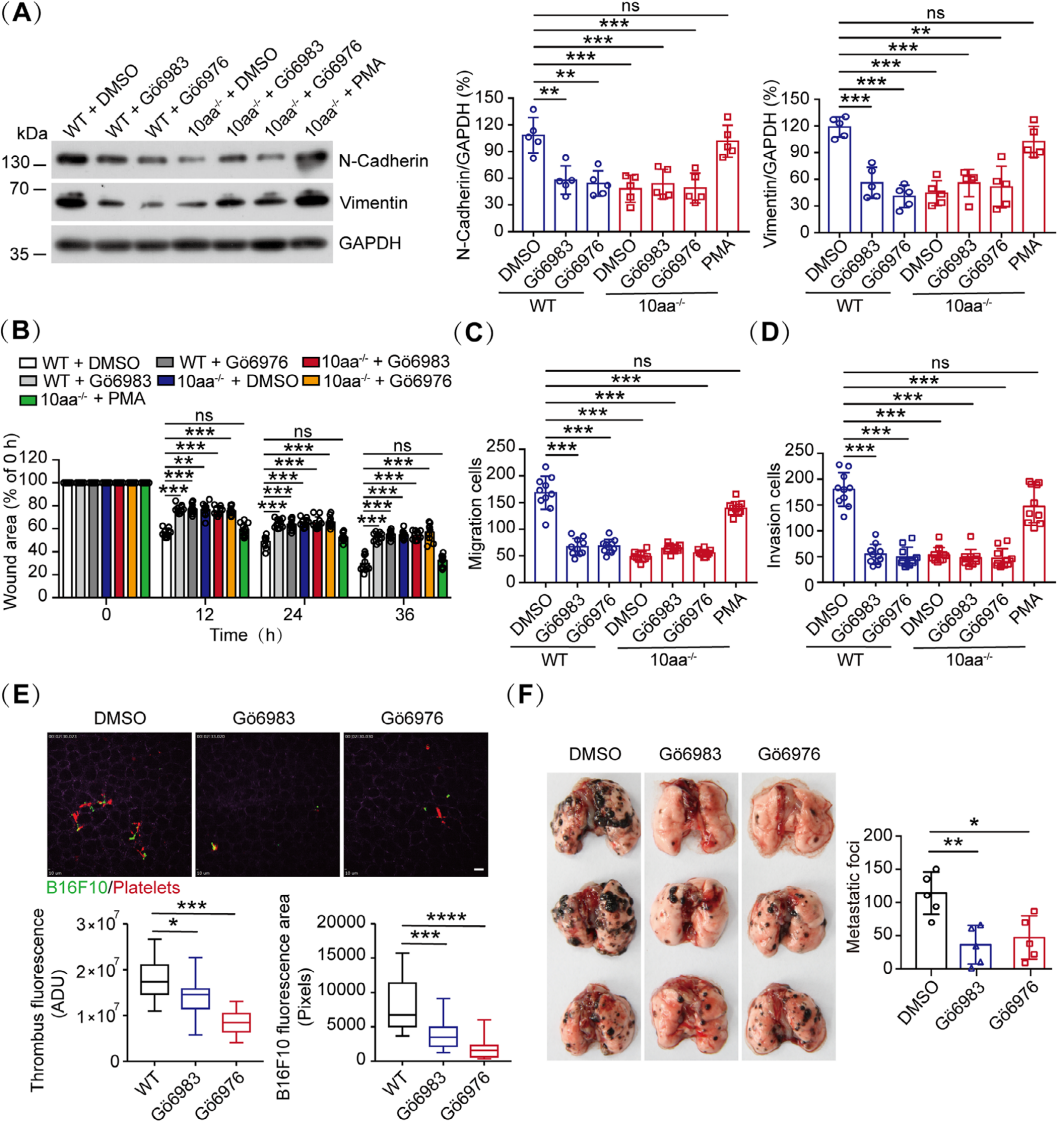
The effect of PKC inhibitors on platelet‐induced B16F10 cell migratory capability and metastasis. (A) Western blotting of N‐cadherin, vimentin, and GAPDH in B16F10 cells treated with WT and 10aa^‐/‐^ platelets (*n* = 5 mice per genotype). (B) Wound‐healing assay of the B16F10 cells incubated with WT and 10aa^‐/‐^ platelets (pretreated by DMSO, Gö6983, Gö6976, or PMA) (*n* = 10 visual fields from five mice of each genotype). (C and D) Migration (C) and invasion (D) of B16F10 cells incubated with washed WT and 10aa^‐/‐^ platelets (pretreated by DMSO, Gö6983, Gö6976, or PMA) (*n* = 10 visual fields from five mice of each genotype). (E) Representative SDIM images and quantification of B16F10‐induced thrombus fluorescence or B16F10 cell area fluorescence in vivo of WT mice (pretreated by Gö6983 and Gö6976) 6 h after intravenous injection of B16F10 cells (*n* = 18 visual fields from three mice of each genotype). Scale bar: 30 µm. (F) The effect of Gö6983 and Gö6976 on tumor pulmonary metastasis in WT mice (*n* = 5 mice per group). One‐way ANOVA followed by Tukey's post hoc test in (A) and (C)–(F). Two‐way ANOVA followed by Bonferroni's post hoc test in (B). Data are shown as mean ± SD (A–D and F) and min to max (E). *p < 0.05, **p < 0.01, ***p < 0.001, and ****p < 0.0001. ns, not significant.

The protein, 14‐3‐3, is an endogenous inhibitor of PKC, and the dissociation of 14‐3‐3 from PKC leads to PKC activation [45]. We have shown in our recent study that the GPIbα cytoplasmic tail promotes PKCα activity through sequestering 14‐3‐3ζ from PKCα [40]. PKCα, the major regulator of α‐ and dense‐granule secretion in platelets, activates αIIbβ3 through inside‐out signaling [46]. Thus, we hypothesized that the GPIbα cytoplasmic tail regulated tumor cell‐induced platelet activation and metastasis through PKCα. To test this, we examined PKCα activity by detecting the phosphorylation of its substrate pleckstrin by western blotting. B16F10 cells significantly promoted WT platelet PKCα activation; however, PKCα activation was inhibited in B16F10 cell‐treated 10aa^−/−^ platelets (Figure 4A). Furthermore, tumor cell‐induced WT platelet P‐selectin (Figures 4B and S5A) and PS exposure (Figures 4C and S5B) and tumor cell–platelet interaction (Figures 4D,E and S6) were significantly inhibited by Gö6983 (an inhibitor of pan‐PKC) and Gö6976 (a specific inhibitor of PKCα and PKCβ1). When the 10aa^‐/‐^ platelets were pretreated with PMA (2‐acetoxy‐1‐methoxypropane, a PKC activator), the B16F10‐induced 10aa^‐/‐^ platelet P‐selectin/PS exposure and B16F10‐platelet interaction were elevated (Figures 4B–E, S5, and S6). Additionally, N‐cadherin, vimentin, and migratory capability were decreased in Gö6983 or Gö6976 pretreated WT platelet‐incubated tumor cells and increased in PMA‐pretreated 10aa^‐/‐^ platelet‐incubated tumor cells (Figures 5A–D and S7). The number of tumor cell–platelet aggregates (Figure 5E and Videos S5–S7) and metastatic nodules (Figure 5F) in the lung were significantly decreased by Gö6983 and Gö6976 in WT mice.

**FIGURE 6:**
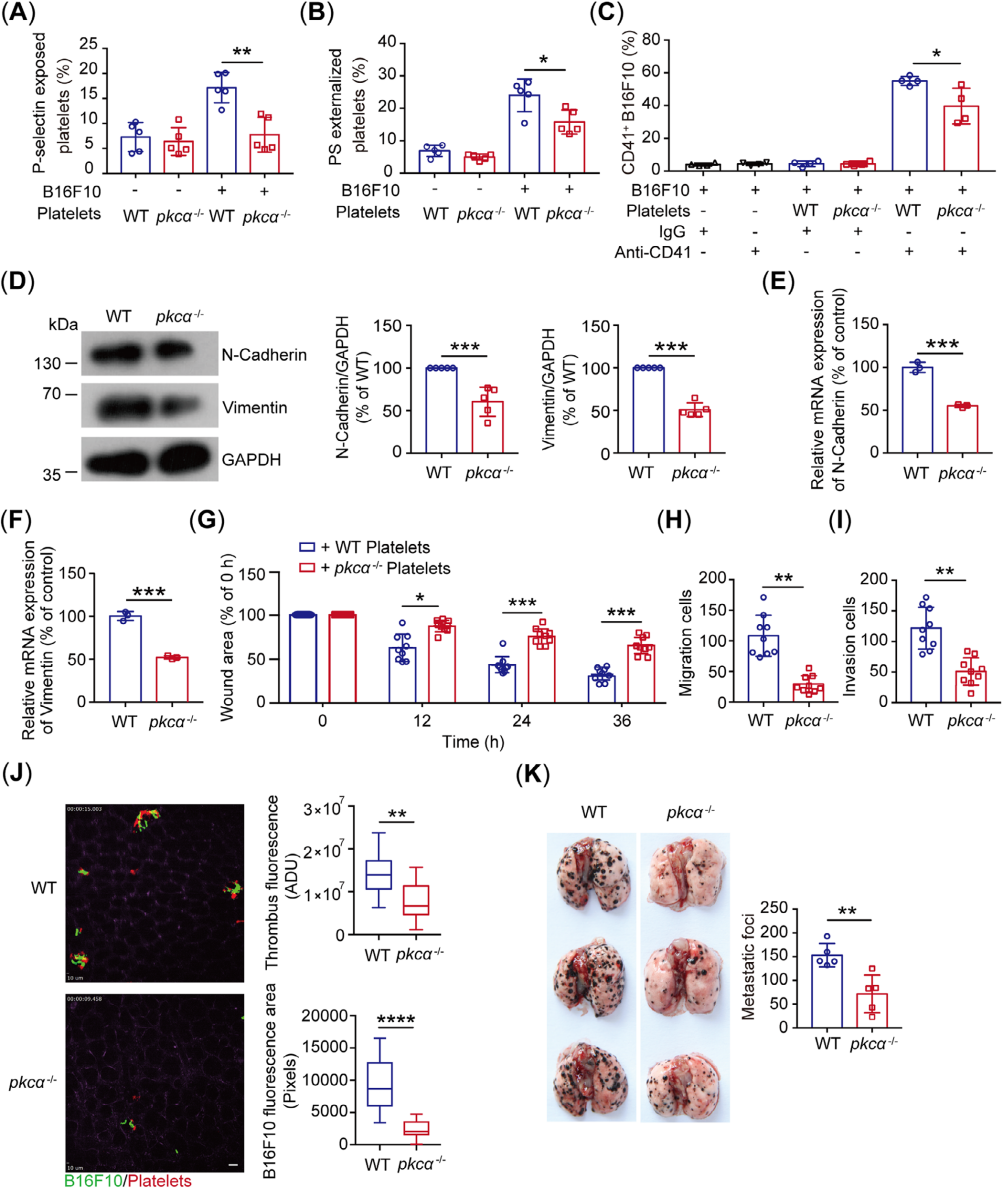
The role of PKCα in tumor‐platelet activation and metastasis. (A and B) B16F10 cell‐induced P‐selectin (A) and PS (B) exposure on washed WT and *pkcα* ^‐/‐^ platelets (*n* = 5 mice per group). PS, phosphatidylserine. (C) The in vitro adhesion of WT and *pkcα* ^‐/‐^ platelets to B16F10 cells was analyzed by flow cytometry using an antiplatelet CD41 antibody (*n* = 5 mice per group). (D) Western blotting of N‐cadherin, vimentin, and GAPDH in B16F10 cells treated with WT and *pkcα* ^‐/‐^ platelets (*n* = 5 independent experiments). (E and F) Relative mRNA expression levels of N‐cadherin (E) and vimentin (F) in B16F10 cells treated with WT and *pkcα^‐/‐^* platelets. (*n* = 3 independent experiments). (G–I) Wound‐healing (G) and transwell assays (H and I) for B16F10 cells after treated with washed WT and *pkcα* ^‐/‐^ platelets (*n* = 9 visual fields from three mice of each genotype). (J) Representative SDIM images (left) of B16F10‐platelet aggregates in the liver of WT and *pkcα* ^‐/‐^ mice 6 h after intravenous injection of B16F10 cells. Quantification (right) of WT or *pkcα* ^‐/‐^ B16F10‐induced thrombus fluorescence or B16F10 cell area fluorescence in vivo 6 h after B16F10 cell intravenous injection (*n* = 21 visual fields from three mice of each genotype). Scale bar: 30 µm. (K) Representative images (left) and number of metastases burden (right) in the lung of WT and *pkcα* ^‐/‐^ mice (*n* = 5 mice per group). One‐way ANOVA followed by Tukey's post hoc test in (A)–(C). Two‐tailed Student's *t*‐test in (D)–(F) and (H)–(K). Two‐way ANOVA followed by Bonferroni's post hoc test in (G). Data are shown as mean ± SD (A–I and K) and min to max (J). *p < 0.05, **p < 0.01, and ***p < 0.001.

Furthermore, we verified the role of PKCα in tumor cell‐induced platelet activation, interaction and metastasis. We found that the *pkcα* ^‐/‐^ platelets, which have normal platelet counts (Figure S8A) and GPIbα expression (Figure S8B) in the platelets, showed lower tumor cell‐induced P‐selectin (Figure 6A) and PS exposure (Figure 6B) and tumor cell–platelet interaction (Figure 6C). Compared with WT platelet‐treated tumor cells, *pkcα* ^‐/‐^ platelet‐treated B16F10 cells exhibited lower migratory capability (Figures 6D–I and S9). Expectedly, the number of tumor cell–platelet aggregates (Figure 6J and Videos S8 and S9) and metastatic nodules in the lung was significantly decreased in *pkcα* ^‐/‐^ mice (Figure 6K).

In summary, these data reveal that the GPIbα cytoplasmic tail deficiency attenuates tumor cell‐induced platelet activation and metastasis by regulating PKCα activation. Therefore, inhibition of platelet activation with the PKCα inhibitor appears to be a novel antimetastasis strategy.

## 3 Discussion

**FIGURE 7:**
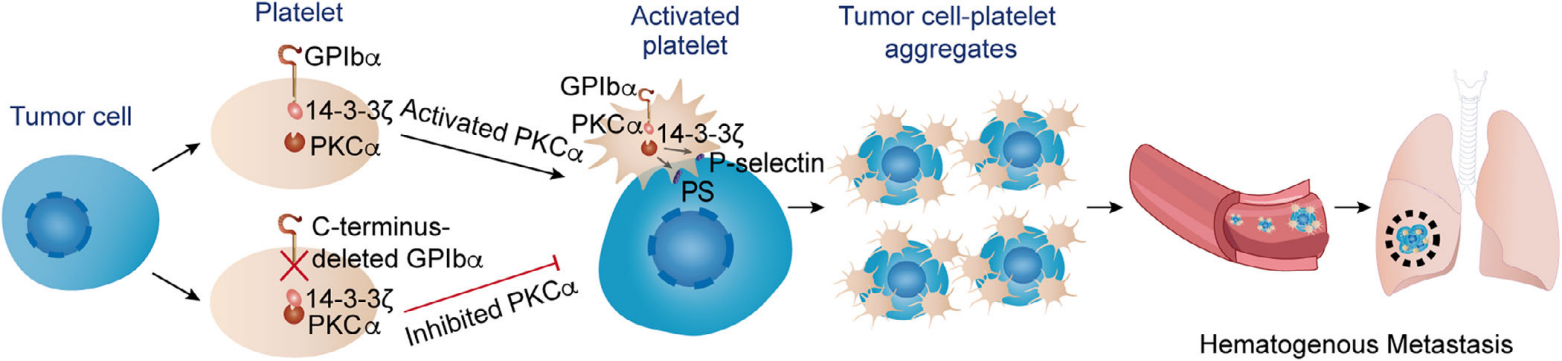
Schematic diagram of the role of platelet GPIbα in metastasis. GPIbα primes and potentiates tumor cell‐induced platelet activation through associating with 14‐3‐3ζ and increasing PKCα activity. GPIbα‐dependent PKCα and platelet activation are required for tumor cell–platelet interaction and hematogenous metastasis. The inhibition of PKCα‐dependent platelet activation by pharmacologic inhibitors or genetic ablation of PKCα attenuates tumor cell–platelet interaction and hematogenous metastasis. Created by Adobe Illustrator.

Although several studies have demonstrated the role of GPIbα in hematogenous metastasis, there are still debates on whether GPIbα favors metastasis or plays a counter role [29, 30, 31, 32, 33]. These studies mainly focused on the GPIbα extracellular domain and the extracellular domain‐mediated potential interaction with endothelial cells and tumor cells [29, 30, 31, 32, 33]. The current study demonstrated the promoting role of GPIbα for metastasis and revealed for the first time that the GPIbα cytoplasmic tail is essential for tumor cell‐induced platelet activation, tumor cell–platelet interaction, and metastasis. Our data also revealed that the GPIbα cytoplasmic tail regulated the tumor cell‐induced platelet PKCα activation, and that the inhibition of PKCα‐dependent platelet activation by pharmacologic inhibitors or genetic ablation of PKCα attenuated the hematogenous metastasis (Figure 7).

Metastasis is the main cause of cancer‐related deaths and remains the biggest challenge in improving cancer prognosis [4]. It has been found that the lack of GPIbα reduced the number of metastatic foci in the lung, but the effect of the severe thrombocytopenia in the mouse model on metastasis was not excluded [29]. We distinguished the role of GPIbα in metastasis by using *Mpl* ^‐/‐^ mice and revealed that GPIbα deficiency inhibited tumor cell‐induced platelet activation and pulmonary metastasis (Figure 1). These data further clarify the role of GPIbα in tumor metastasis.

It has been well accepted that platelet–tumor cell aggregates are prerequisites for hematogenous metastasis [47]. Tumor cells activate platelets to form stable platelet–tumor cell aggregates [8]. Tumor cells release soluble mediators, such as ADP, thromboxane A2 (TXA2), thrombin, and high‐mobility group box1, to activate local platelets by engaging with their receptors on platelets [8, 48, 49, 50]. Tumor cells also activate platelets through direct contact. For example, various tumor cells express the counter‐receptor ADAM9 (a disintegrin and metalloproteinase 9) for platelet integrin α6β1 and bind to α6β1 to activate platelets [51]. Colon and breast cancer cells express the counter‐receptor Galectin‐3 for platelet GPVI and activate platelets through GPVI‐mediated signaling [52]. We have recently shown that the GPIbα cytoplasmic tail is required for general platelet activation, including ADP‐P2Y, U46619 (a TXA2 mimic)‐thromboxane A2 (TP) receptor, collagen/collagen‐related polypeptide‐GPVI, and protease‐activated receptor 4 (PAR4)‐activating peptide‐PAR4‐initiated signaling [40]. Thus, the GPIbα cytoplasmic tail may facilitate platelet activation through multiple pathways induced by tumor cells. Indeed, the 10aa^‐/‐^ platelets could not be easily activated by tumor cells in vitro and in vivo. This is also true for *Gp1ba* ^‐/‐^ platelets. Notably, 10aa^‐/‐^ and *Gp1ba* ^‐/‐^ platelets were in more resting states, reflected by the decreased surface expression levels of P‐selectin and PS, suggesting that the GPIbα cytoplasmic tail primes platelets to be activated by tumor cells. To the best of our knowledge, this is the first study to show that the basal surface expression level of P‐selectin was decreased in resting *Gp1ba^‐/‐^* platelets. P‐selectin is a specific marker of platelet α‐granules [53]. Recently, we found that the deletion of the cytoplasmic tail of GPIbα in mouse platelets (10aa^‐/‐^) decreased the PKC substrate phosphorylation level and platelet α‐granule secretion [40]. Therefore, the decreased surface P‐selectin expression in resting *Gp1ba^‐/‐^* platelets may be associated with decreased basal PKCα activity. However, the mechanism still needs to be further investigated in the future.

Activated platelets form more stable adhesions with tumor cells. At the molecular level, P‐selectin, expressed on the activated platelets, is important for platelet–tumor cell interaction because P‐selectin‐deficient platelets have decreased interaction with tumor cells [37, 54, 55]. Thus, platelet–tumor cell adhesion was decreased for 10aa^‐/‐^ platelets both in vitro and in vivo. However, Jain et al. [29] did not detect a difference in platelet–tumor cell interaction between WT and GPIbα‐deficient platelets. This discrepancy may be explained by the neutralization of decreased adhered platelets to tumor cells by the increased fluorescent signal of CD41 on GPIbα‐deficient platelets due to their larger size [34, 50]. Besides P‐selectin, the extracellular domain of platelet GPIbα also supports experimental metastasis [29]. However, because the platelet GPIbα extracellular domain does not directly mediate the heterotypic interactions with B16F10 cells [29] and deletion of the GPIbα cytoplasmic tail does not affect the VWF binding or antibody binding functions of the GPIbα extracellular domain [40, 56], the GPIbα cytoplasmic tail is unlikely to regulate platelet–B16F10 interaction through affecting the binding of the GPIbα extracellular domain to B16F10 cells. The decreased adhesion not only reduced the cloak of platelets on tumor cells but also diminished the migration and invasion of B16F10 cells, leading to impaired experimental metastasis. Similarly, the pulmonary metastasis was also reduced in the GPIbα C‐terminal six residue‐deficient mice, but unfortunately, no statistical significance was found between the groups of WT and GPIbα truncated mice [29].

The PKC signaling pathway is important to platelet function and plays an essential role in platelet adhesion, secretion, and other functions [57]. PKC also participates in the thrombosis process in vivo [58]. PKC has a variety of subtypes, including α, β, σ and θ subtypes expressed in platelets, while the PKCα subtype is the main regulator of the secretion of α and dense granule in platelets and can activate αIIbβ3 through the internal and external signaling pathways [46, 57, 58, 59]. Recently, we found that the GPIbα cytoplasmic tail promotes general platelet activation by sequestering 14‐3‐3ζ from PKCα to potentiate PKCα activity [40]. Additionally, PKCα is also involved in hyperglycemia‐induced platelet activation to promote metastasis [11]. Here, we demonstrated that the GPIbα cytoplasmic tail regulated the tumor cell‐induced platelet PKCα activation, and the inhibition of PKCα‐dependent platelet activation effectively prevented metastasis. These findings explain the mechanism underlying the action of the GPIbα cytoplasmic tail and, more importantly, experimentally demonstrate that the inhibition of PKCα‐dependent platelet activation is a feasible strategy for diminishing metastasis. PKC has served as an attractive target for anticancer drug development because it regulates a large number of enzymes and transcription factors involved in carcinogenic signaling [60]. PKCα in cancer cells positively regulates their motility and migration [61]. Thus, inhibition of PKCα may permit a double‐strike attack on metastasis, targeting not only PKCα in tumor cells but also that in platelets. It is noteworthy that PKCβ1 is also expressed in platelets [46] and can be inhibited by Gö6976. The role of PKCβ1 in metastasis warrants further investigation. For the future study, spontaneous metastasis models and additional tumor cell lines will be used to validate the role of GPIbα and GPIbα‐regulated PKCα activation in metastasis.

In summary, our data clarify the role of GPIbα in experimental metastasis and reveal for the first time that GPIbα promotes experimental metastasis through priming and potentiation of platelet activation via its cytoplasmic tail. Targeting the platelet GPIbα or the GPIbα cytoplasmic tail may inhibit the motility and migration of malignant disease. Additionally, our study highlights the function of PKCα in GPIbα‐regulated platelet activation and metastasis and strengthens the metastasis‐promoting activity of PKCα from the perspective of platelets, which may have therapeutic implications for suppressing tumor metastasis.

## 4 Materials and Methods

### 4.1 Cell Lines and Animals

Mouse cell line B16F10 (CL‐0319) was kindly provided by Wuhan Pricella. C57BL/6J mice of the WT strain were acquired from JOINN Laboratories (Suzhou, China). *Gp1ba* ^‐/‐^ [32] and 10aa^‐/‐^ [38] mice were described previously. C57B6L/6JGpt‐*Prkca* ^em1Cd5428^/Gpt mice (*PKC*α^‐/‐^, T027858) were purchased from GemPharmatech Co, Ltd (Nanjing, China). *Mpl* ^‐/‐^ mice were provided by Junping Wang (Third Military Medical University, Chongqing, China). Furthermore, animals utilized in this work were provided sustenance inside the specific pathogen‐free (SPF) animal facility at Soochow University. Additionally, all animal experiments comply with the ARRIVE guidelines (U.K. Animals (Scientific Procedures) Act, 1986 and EU Directive 2010/63/EU for animal experiments). Mice 6–12‐week‐old were used in this study and groups of female and male mice were balanced in all animal experiments.

### 4.2 Statistics Analysis

GraphPad Prism 8 software was used for statistical analysis. Shapiro–Wilk test was done for normality, and Brown–Forsythe test was done for variance. For all normally distributed data, a single variant numeric data were analyzed using one‐way ANOVA, and multiple variants numeric data were analyzed using two‐way ANOVA. An unpaired two‐tailed Student's *t*‐test was used for comparisons between two groups. Differences were considered significant at *p < 0.05, **p < 0.01, and ***p < 0.001.

More detailed Materials and Methods can be found in the Supporting Information.

## Author Contributions

Conceptualization, design of the study, and writing: K. Z., K. D., and R. Y. Methodology, investigation, validation, and formal analysis: K. Z., Q. L., Y. X., C. S., J. W., Y. S. X. G. M. Y., Y. L., and S.Z. Methodology, resources, and formal analysis: L. Z., C. L., K. M. S., W. X., and R. H. Conceptualization, supervision, project administration, and funding acquisition: K. D. and R. Y. All authors have read and approved the final manuscript.

## Ethics Statement

All animal protocols were approved by the Institutional Laboratory Animal Care and Use Committee of Soochow University (No. SDFYY2022495). All animal experiments comply with the ARRIVE guidelines (U.K. Animals (Scientific Procedures) Act, 1986 and EU Directive 2010/63/EU for animal experiments).

## Conflicts of Interest

The authors declare no conflicts of interest.

## Supporting information



Supporting Information

Supporting Information

Supporting Information

Supporting Information

Supporting Information

Supporting Information

Supporting Information

Supporting Information

Supporting Information

Supporting Information

## Data Availability

All the data supporting the findings of this study are available within the article and its supporting information files or from the corresponding authors upon reasonable request.
