# Supplementary material for: Glycoprotein Ibα‐Dependent Platelet Activation is Essential for Tumor Cell–Platelet Interaction and Experimental Metastasis
Source: MedComm (2020). 2025 Jun 9;6(6):e70217. doi: 10.1002/mco2.70217 (PMC12146664; doi:10.1002/mco2.70217)

Supplementary Information

Glycoprotein Ibα-dependent platelet activation is essential for tumor cell-platelet interaction and experimental metastasis

Kangxi Zhou#; Qing Li#; Yue Xia#; Chenglin Sun#; Jing Wang; Yueyue Sun; Xinxin Ge; Mengnan Yang; Yu Li; Sai Zhang; Lili Zhao; Chunliang Liu; Khan Muhammad Shoaib; Weiling Xiao; Renping Hu; Kesheng Dai* and Rong Yan*

Jiangsu Institute of Hematology, The First Affiliated Hospital of Soochow University, Cyrus Tang Medical Institute, Suzhou Medical College, Soochow University, NHC Key Laboratory of Thrombosis and Hemostasis, National Clinical Research Center for Hematological Diseases, Suzhou, Jiangsu 215006, China.

#These authors contributed equally: Kangxi Zhou, Qing Li, Yue Xia, Chenglin Sun.

***Correspondence**

Kesheng Dai, Jiangsu Institute of Hematology, The First Affiliated Hospital of Soochow University, Cyrus Tang Medical Institute, Suzhou Medical College, Soochow University, NHC Key Laboratory of Thrombosis and Hemostasis, National Clinical Research Center for Hematological Diseases, Suzhou, Jiangsu 215006, China.

Email: kdai@suda.edu.cn

Rong Yan, Jiangsu Institute of Hematology, The First Affiliated Hospital of Soochow

University, Cyrus Tang Medical Institute, Suzhou Medical College, Soochow University, NHC Key Laboratory of Thrombosis and Hemostasis, National Clinical Research Center for Hematological Diseases, Suzhou, Jiangsu 215006, China.

Email: yanrongbaobao@163.com

MATERIALS AND METHODS

Reagents: Mouse cell line B16F10 was obtained from American Type Culture Collection (ATCC). FITC-conjugated rat anti-mouse P-selectin (clone Wug.E9, D200), FITC-conjugated rat anti-mouse GPIbα (clone Xia. G5, M040-1), and Dylight 649-conjugated anti-GPIbβ (x649) antibodies were obtained from Emfret Analytics (Eibelstadt, Germany). Rat IgG (A0192), rabbit IgG (A0208) and HRP-conjugated goat anti-mouse (A0216) were purchased from Beyotime Biotechnology (Shanghai, China). The BCA protein assay kit (23225) was purchased from Thermo Fisher Scientific (Rockford, IL, USA). PE-conjugated rat anti-mouse CD41 antibodies (clone MWReg30, 133906) were purchased from Biolegend (San Diego, CA, USA). Antibodies against N-cadherin (66219-1-Ig) and vimentin (10366-1-AP) were obtained from Proteintech (XYbscience, USA). Rabbit antibodies against GAPDH (5174), phospho-PKC substrate (2261), and 10 × cell lysis buffer (9803) were sourced from Cell Signaling Technology (Beverly, MA, USA). Gö6983 (S2911), Gö6976 (S7119), and phorbol ester (PMA) (S7791) were purchased from Selleck (Houston, TX, USA).

Hematologic analysis: Murine whole blood anti-coagulated with a 1/7 volume of acid-citrate-dextrose (ACD, 1.5% citric acid, 2.0% D-glucose, and 2.5% trisodium citrate) was collected from the postorbital veins. Mindray BC-5000vet Hematologic Analyzer (Mindray Corporation, Shenzhen, China) was used for platelet count.

Platelet preparation: Murine whole blood, anti-coagulated with 1/7 volume of ACD, was collected from postorbital veins or the inferior vena cava. Platelets were washed

with the buffer (0.013 M trisodium citrate, 0.033 M D-glucose, 0.123 M NaCl , pH 6.5) and resuspended in Modified Tyrode's buffer (MTB, 5.5 mM D-glucose, 12 mM $NaHCO_3$, 150 mM NaCl, 1 mM $CaCl_2$, 1 mM $MgCl_2$, 2.5 mM KCl, 2.5 mM Hepes (N-2-hydroxyethylpiperazine-N'-2-ethanesulfonic acid), pH 7.4). The concentration of platelets was 3×10^8 ml^{-1}, and the resuspended platelets were allowed to stay at room temperature (RT) for 1-2 hours (h). Murine platelet-rich plasma (PRP) was obtained by 100 × g centrifugation of murine whole blood (anti-coagulated with 1/9 volume of 3.8% trisodium citrate).

Flow cytometry: Murine platelet-rich plasma (PRP) from WT, *Gp1ba*$^{-/-}$, 10aa$^{-/-}$ and *Mpl*$^{-/-}$ mice were double-labeled by PE-conjugated anti-mouse CD41 (Biolegend, clone MWReg30, 133906, 2 μg/mL) and FITC-conjugated anti-mouse GPIbα (Emfret Analytics, clone Xia. G5, M040-1, 5 μg/mL) antibodies at RT for 30 min. For detecting the basal levels of P-selectin and PS exposure on WT, *Gp1ba*$^{-/-}$, 10aa$^{-/-}$ and *Mpl*$^{-/-}$ PRP were double-labeled by APC-conjugated anti-mouse CD41 (Biolegend, clone MWReg30, 133914, 2 μg/mL) and FITC-conjugated an anti-mouse P-selectin antibody (Emfret Analytics, clone Wug.E9, D200, 5 μg/mL) or FITC-conjugated lactadherin (Haematologic Technologies, 10 μg/mL). Data normalization: mean fluorescence intensity (MFI) of per sample/average of WT. For detecting platelet activation, washed murine platelets were stimulated with B16F10 (20:1). P-selectin exposure was detected with anti-mouse P-selectin antibody (Emfret Analytics, clone Wug.E9, D200, 5 μg/mL). PS externalization of mouse platelets was detected by FITC-labeled lactadherin (Haematologic Technologies, 10 μg/mL).

Western blotting: 1 × 10^5 B16F10 cells were incubated with washed sterile WT (pre-treated by DMSO, Gö6983, Gö6976 or not), 10aa$^{-/-}$ (pre-treated by DMSO or PMA) or *pkca*$^{-/-}$ platelets (1:20) in 6-well plates for 24 h. B16F10 cells were separated by centrifuge at 64 × g for 5 min, and lysed with a 1/9 volume of 10 × lysis buffer (CST) containing phenylmethylsulfonyl fluoride (1 mM) and protease inhibitor cocktail on ice for 30 min. Protein concentration was measured by the BCA protein assay kit (Thermo Fish Scientific) and adjusted to the same level by lysis buffer. Proteins were separated by SDS-PAGE and immunoblotted with specific antibodies. Quantification was performed with ImageJ software (NIH).

Cell migration and invasion assays: A wound healing assay was used to measure the migration of differently treated B16F10 cells. After the cell proliferation in the 6-well plate reached approximately 90% confluence, a 10 μL pipette tip was used to evenly scrape the cell layer to form a scratch. After that, the scraped cell debris was gently removed with PBS. Then, serum-free medium was added, and pictures of the scratch were immediately photographed under a microscope. Then, B16F10 cells were cultured with wild-type, *pkcα*$^{-/-}$ or 10aa$^{-/-}$ platelets. Wild-type platelets pretreated with in the presence or absence of Gö6976 (5 μM) or Gö6983 (5 μM), or 10aa$^{-/-}$ platelets were pretreated with in the presence or absence of PMA (2 nM) at 37°C for 10 minutes. Every 12 h, pictures of the cell scratches were taken again, and the wound area was analyzed using ImageJ software.

In addition, the classic Transwell chamber (membrane with 8-μm pore size, Corning Life Sciences, United States) was used to further evaluate the migration or invasion (with Matrigel) of cancer cells. B16F10 cells were cultured with washed

wild-type (presence or absence of 5 μM Gö6976, 5 μM Gö6983), *pkcα*$^{-/-}$, or 10aa$^{-/-}$ (presence or absence of 2 nM PMA) platelets (1:1) in 200 μL of serum-free medium and then plated in the upper chamber. After 36 h, the B16F10 cells were fixed with 4% paraformaldehyde for 30 min and stained with 0.1% crystal violet for 15-20 min. Under the chamber membrane cells were counted and photographed through an inverted microscope.

Quantitative real-time polymerase chain reaction: The total RNA of B16F10 cells (after pretreatment with WT or *pkcα*$^{-/-}$ platelets for 24 h), washed WT and *Gp1ba*$^{-/-}$ platelets were extracted by TRIzol reagent (Life Sciences; Solarbio, Inc.). Fast quant RT kit (Vazyme Biotech Co., Ltd., Nanjing, China) was used for reverse transcription according to the manufacturer's protocol. The mixture volume of quantitative real-time polymerase chain reaction (qPCR) was 20 μL, including 7.8 μL RNAse-free H_2O, 0.2 μL primers per mix (100 μM each primer), 2 μL cDNA, and 10 μL SYBR green mix. PCR program on ABI 7500 (Applied Biosystems Inc. USA): 95°C for 2 min, 1 cycle; 40 cycles of 95°C for 10 s, 56°C for 30 s, 72°C for 30 s. Specific primers were designed by gene runner software and were synthesized by Shanghai Sangon Biotechnology Co., Ltd (Shanghai, China). The specific primers: N-cadherin-Forward:GCCAACCTAACTGTCACGGA; N-cadherin-Reverse:GGGTCTGTCAGGATGGCAAA; Vimentin-Forward:AGCAGTATGAAAGCGTGGCT; Vimentin-Reverse:ACCTGTCTCCGGTACTCGTT; P-selectin-Forward:CGAGCTGCAATGTTTGGCTT; P-selectin-

Reverse:GCAATTGGGTGCATACAGGC; GAPDH-

Forward:AGGTCGGTGTGAACGGATTTG; GAPDH-

Reverse:GGGGTCGTTGATGGCAACA. The relative quantification of gene expression was calculated using the 2-ΔΔCt method. The targeted gene expression was presented as a proportion of control gene (GAPDH).

Observing tumor cell-platelet interaction by state-of-the-art spinning disk intravital microscopy (SDIM) in vivo: Wild-type, $10aa^{-/-}$, or $pkc\alpha^{-/-}$ mice were injected with 1×10^5 B16F10 cells (in 100 μL PBS) via the lateral tail veins. In inhibition experiments, C57BL/6J mice were i.v. pre-injected with Gö6983 or Gö6976 (0.5 μg/g) 10 min before the injection of B16F10 cells (1×10^5 in 100 μL PBS). Circulating platelets are labeled by Dylight 649-conjugated anti-GPIbβ antibody (0.075 μg/g), and the breathing of anesthetized mice was assisted by the ventilator (ALC-V8S-631, ALCBIO). Lung imaging was monitored in real time by bright field and spinning disk intravital confocal microscopy (SDIM, VIVO Intravital Imaging System, Intelligent Imaging Innovations) using a sCMOS camera (ORCA Flash 4.0, Hamamatsu).

Experimental lung metastasis: Wild-type, $Gp1ba^{-/-}$,$10aa^{-/-}$, $Mpl^{-/-}$ or $pkc\alpha^{-/-}$ mice were injected with 1×10^5 B16F10 cells (in 100 μL PBS) via the lateral tail veins. In inhibition experiments, C57BL/6J mice were i.v. pre-injected with Gö6983 or Gö6976 (0.5 μg/g) 10 min before the injection of B16F10 cells (1×10^5 in 100 μL PBS). Mice were sacrificed two weeks after injection and the lungs were isolated. Surface metastatic foci in the lung were counted under a dissecting microscope. For the

hematoxylin and eosin (H&E) staining of the lung cryosections, the lungs were immediately embedded in Tissue-Tek OCT and stored at -80°C. Lung cryosections were fixed in 10% paraformaldehyde and stained with H&E. Metastatic foci were examined by an optical microscope (CX31, Olympus, Tokyo, Japan) with a 10 × objective lens.

Platelet aggregation and activation induced by B16F10 cells: Washed wild-type and $10aa^{-/-}$ platelets (4.5 × 10^8/mL, 200 μL) were stimulated with B16F10 cells (3 × 10^6/mL, 100 μL). Platelet aggregation was recorded in the Chrono-Log aggregometer over 5 min. For B16F10 cell-induced platelet activation, washed wild-type and $10aa^{-/-}$ platelets (2 × 10^7/mL, 100 μL) were incubated with B16F10 cells (1 × 10^6/mL, 100 μL) in the presence or absence of Gö6983(5 μM), Gö6976 (5 μM) or PMA (2 nM) at RT for 20 min. Platelet P-selectin and PS exposure were detected by flow cytometry.

Platelet clearance in vivo*:* For B16F10 cell-induced mouse platelet clearance, 1 × 10^6 B16F10 cells (in 100 μL PBS) were i.v. injected slowly into wild-type or $10aa^{-/-}$ mice via the lateral tail veins. The whole blood was collected and platelet counts were determined at 5 min, 30 min, 2 h, 24 h, 48 h and 72 h. Apoptotic markers were detected in platelets in PRP from whole blood collected 5 min after peptide or PMA injection by flow cytometry.

Establishment of B16F10 overexpressing Green Fluorescent Protein (GFP): HBLV-luc-GFP-PURO lentivirus was packed by Hanbio Technology (Shanghai, China). The B16F10 cell line was infected with recombinant HBLV-luc-GFP-PURO

lentivirus to generate B16F10-luc-GFP-PURO cell line (designated B16F10-GFP). The target cells were digested into a single cell suspension and cultured in a 10 cm plate one day before infection. When the coverage reached 60%, cells were infected with lentivirus at a multiplicity of infection of 10 and 8 μg/mL polybrene was added to the supernatant. The culture medium was refreshed 8 h after infection. For stable expression of green fluorescence, the positive cells were sorted by flow cytometry (Beckman Moflo XDP). Sorting was carried out for another time after two weeks' recovery. Then B16F10-GFP cells were confirmed by flow cytometry.

The adhesion of platelets to B16F10 cells in vitro: Washed wild-type and $10aa^{-/-}$ platelets (3×10^8/mL, 30 μL) were incubated with B16F10 cells (1.0×10^6/mL, 45 μL) in the presence or absence of Gö6983(5 μM), Gö6976 (5 μM) or PMA (1 nM) at RT for 20 min. Platelet-adhered B16F10 cells were labeled by PE-conjugated anti-mouse CD41 antibody (5 μg/mL) and measured by flow cytometry.

To characterize the interaction of platelets with B16F10 cells by microscopy, GFP-B16F10 cells (1×10^5 in 2 mL) were seeded in a glass-bottom cell culture dish (20 mm) and grown until confluent. GFP-B16F10 cells were washed by 1640 medium (with 10% fetal bovine serum) three times and incubated with washed wild-type and $10aa^{-/-}$ platelets (1.5×10^7 in 1 mL 1640 medium) in the presence or absence of Gö6983(5 μM), Gö6976 (5 μM) or PMA (1 nM) at 37°C for 1 h. After washed thoroughly, platelets were labeled by APC-conjugated anti-mouse CD41 antibody (clone MWReg30, 5 μg/mL). Cell nuclei were stained with 5 μg/mL DAPI. Cells were observed with a LEICA TCS SP8 confocal microscope with a 63 × oil immersion lens.

The adhesion of platelets to B16F10 cells in vivo: GFP-B16F10 cells (1×10^5 in 100 μL PBS) were i.v. injected into wild-type or $10aa^{-/-}$ mice. After 6 h, mice were sacrificed and the lungs were immediately excised. Lung cryosections were fixed in ice-cold acetone and subsequently blocked in 5% BSA/PBS overnight at RT. APC anti-mouse CD41 (clone MWReg30, 5 μg/mL) was incubated at RT for 1 h. Cell nuclei were stained with 5 μg/mL DAPI. The cryosections were observed with a LEICA TCS SP8 confocal microscope with a 63 × oil immersion lens.

Supplementary Figures and Legends

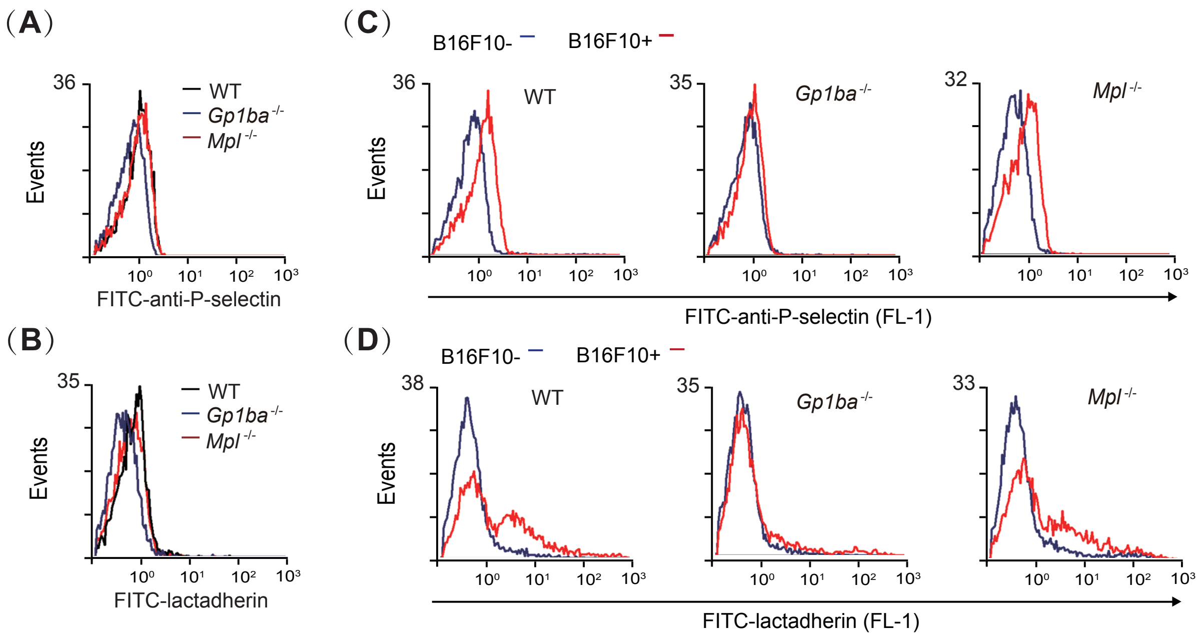

Figure S1. Representative flow cytometry histograms.

A and **B**. Representative flow cytometry histograms of basal P-selectin (**A**) and PS (**B**) levels on resting WT, *Gp1ba*$^{-/-}$ and *Mpl*$^{-/-}$ platelets in PRP. **C** and **D**, Representative flow cytometry histograms of B16F10 cells-induced P-selectin (**C**) and PS (**D**) exposure on WT, *Gp1ba*$^{-/-}$ and *Mpl*$^{-/-}$ platelets.

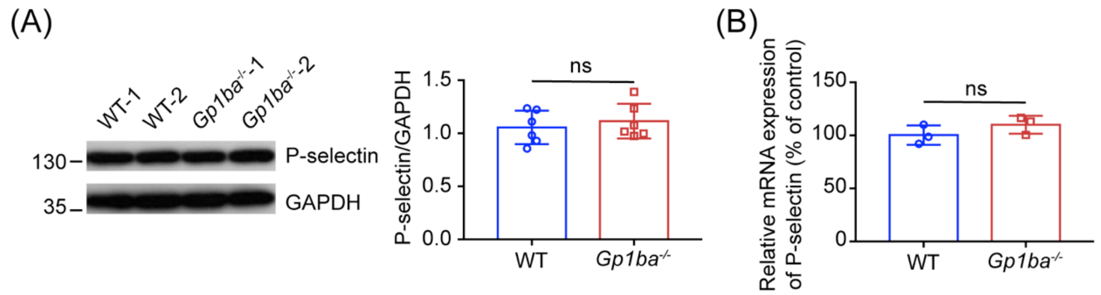

Figure S2. Protein and mRNA expression levels of *Gp1ba*$^{-/-}$ platelets. A, Protein levels of P-selectin in *Gp1ba*$^{-/-}$ platelets (n = 6 independent experiments). **B,** mRNA expression levels of P-selectin in *Gp1ba*$^{-/-}$ platelets (n = 3 independent experiments). Data are shown as mean ± SD. ns, no significant.

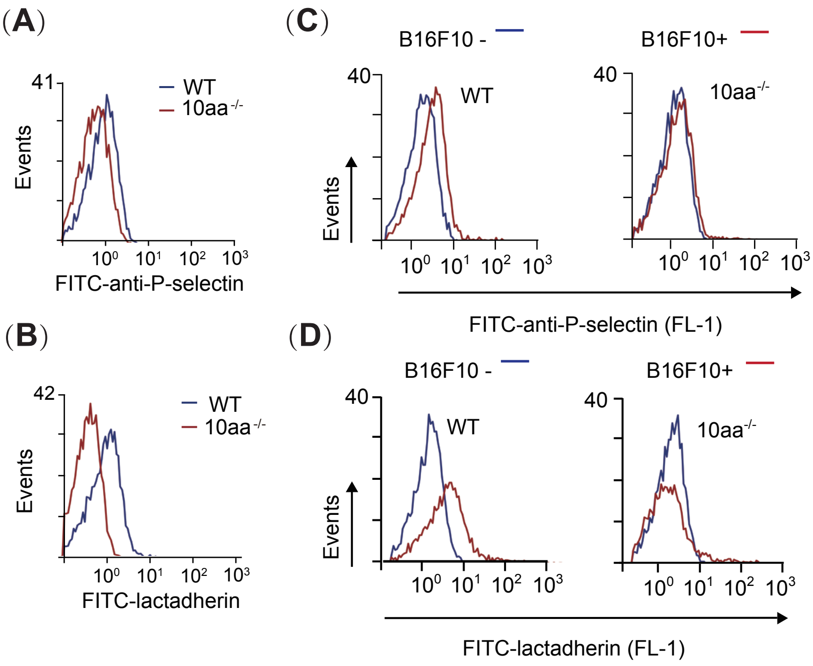

Figure S3. Representative flow cytometry histograms.

A and **B**. Representative flow cytometry histograms of basal P-selectin (**A**) and PS (**B**) levels on resting WT and $10aa^{-/-}$ platelets in PRP. **C** and **D**, Representative flow cytometry histograms of B16F10 cells-induced P-selectin (**C**) and PS (**D**) exposure on WT and $10aa^{-/-}$ platelets.

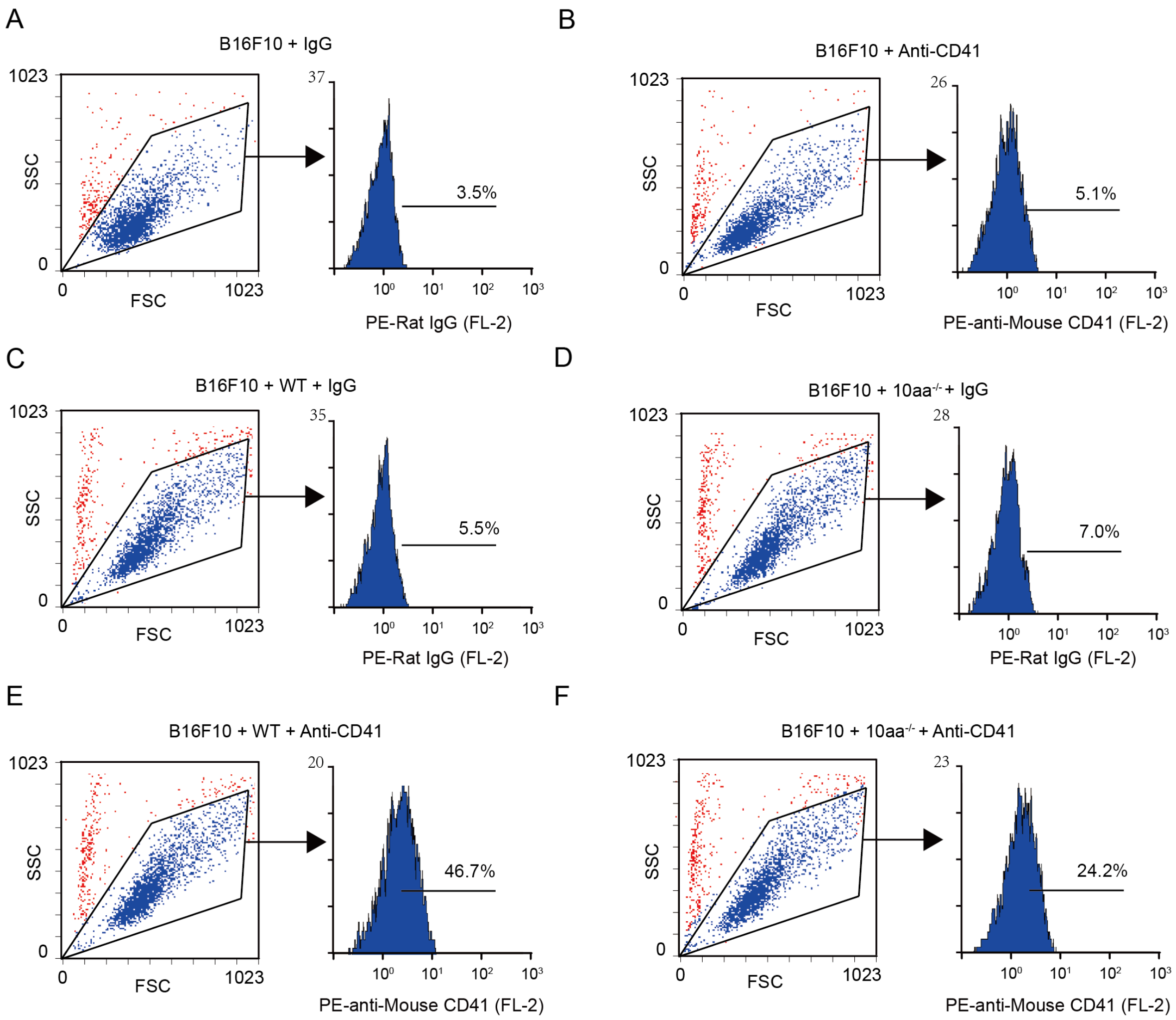

Figure S4. Flow cytometry gating strategies

A-F, Representative flow cytometry images of CD41^{+} B16F10 cell percentages treated with WT and 10aa$^{-/-}$ platelets.

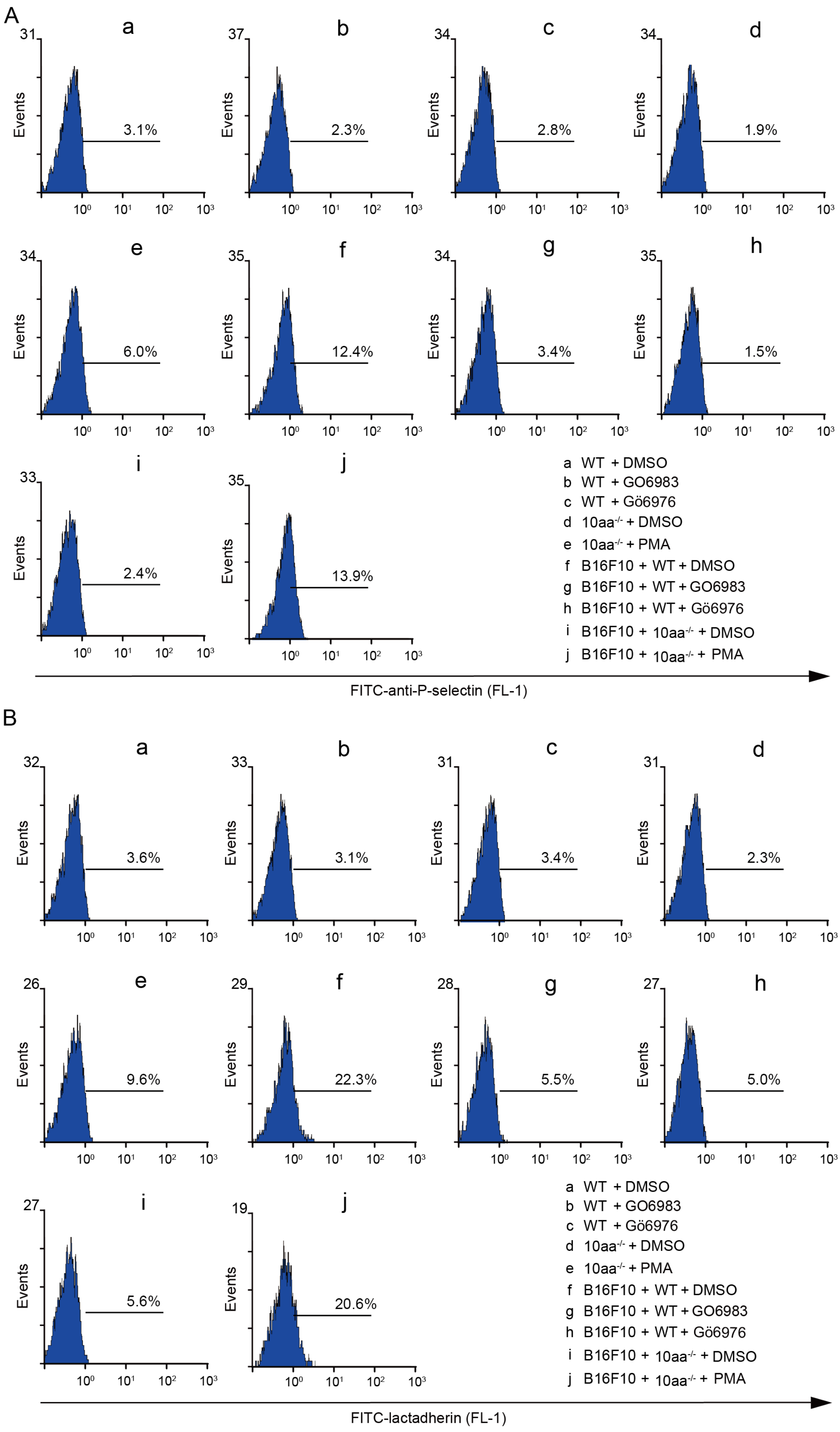
A
a
b
c
d
e
f
g
h
i
j
Events
3.1%
2.3%
2.8%
1.9%
6.0%
12.4%
3.4%
1.5%
2.4%
13.9%
a WT + DMSO
b WT + GO6983
c WT + Gö6976
d 10aa-/- + DMSO
e 10aa-/- + PMA
f B16F10 + WT + DMSO
g B16F10 + WT + GO6983
h B16F10 + WT + Gö6976
i B16F10 + 10aa-/- + DMSO
j B16F10 + 10aa-/- + PMA
FITC-anti-P-selectin (FL-1)
B
3.6%
3.1%
3.4%
2.3%
9.6%
22.3%
5.5%
5.0%
5.6%
20.6%
a WT + DMSO
b WT + GO6983
c WT + Gö6976
d 10aa-/- + DMSO
e 10aa-/- + PMA
f B16F10 + WT + DMSO
g B16F10 + WT + GO6983
h B16F10 + WT + Gö6976
i B16F10 + 10aa-/- + DMSO
j B16F10 + 10aa-/- + PMA
FITC-lactadherin (FL-1)

Figure S5. Representative flow cytometry images

Representative flow cytometry images of B16F10 cell-induced washed WT and $10aa^{-/-}$ platelet P-selectin (**A**) and PS exposure (**B**), pre-treated with Gö6983, Gö6976 or PMA.

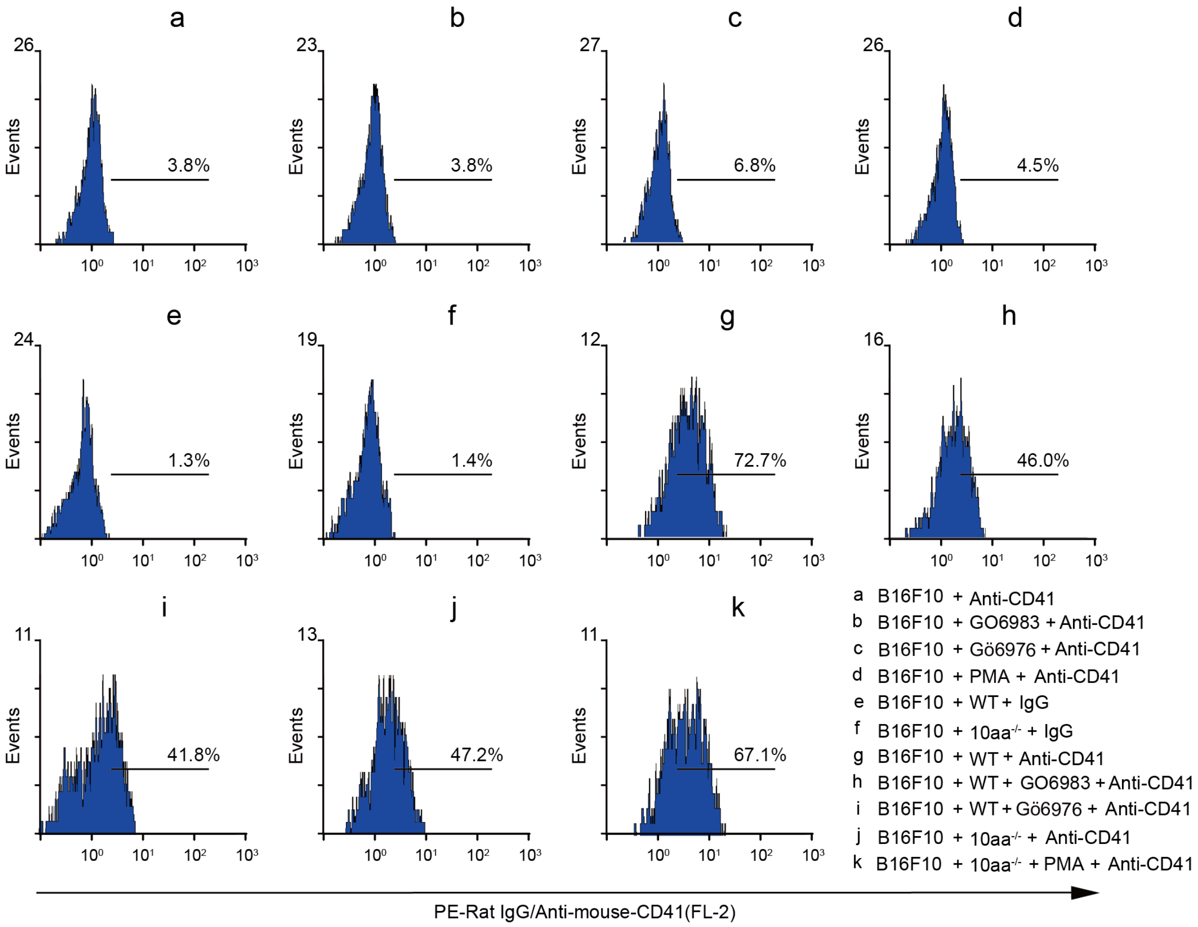

Figure S6. Representative flow cytometry images

Representative flow cytometry images of adhesion of B16F10 cells with WT and $10aa^{-/-}$ platelets pre-treated with Gö6983, Gö6976 and PMA.

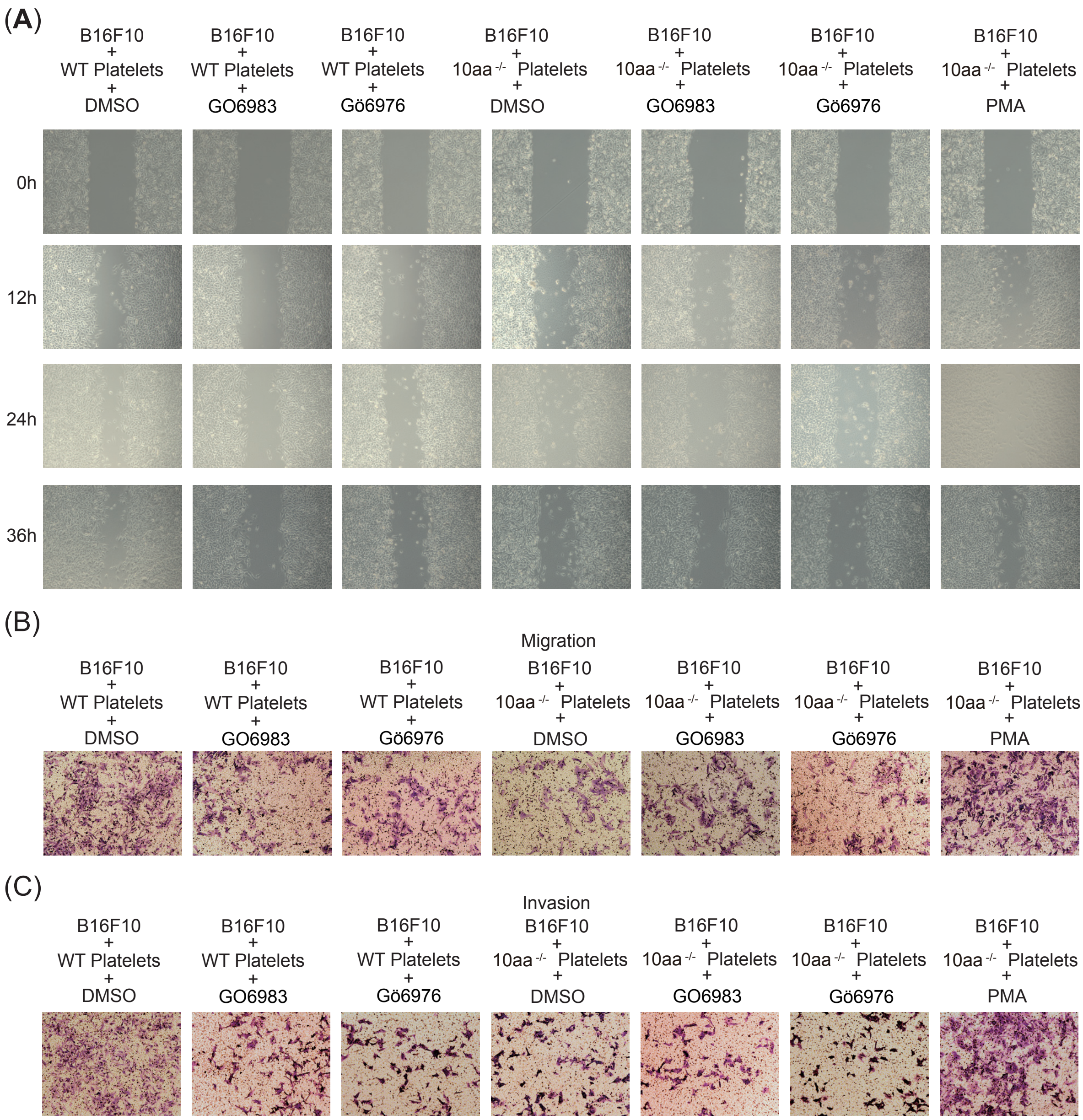

Figure S7. Representative wound-healing and transwell assays images

A, Representative wound-healing images of B16-F10 cells incubated with washed WT and 10aa$^{-/-}$ platelets (pre-treated with DMSO, Gö6983, Gö6976 or PMA). **B and C**, Representative invasion (**B**) or migration (**C**) images of B16-F10 cells were incubated with washed WT and 10aa$^{-/-}$ platelets (pre-treated with DMSO, Gö6983, Gö6976 or PMA).

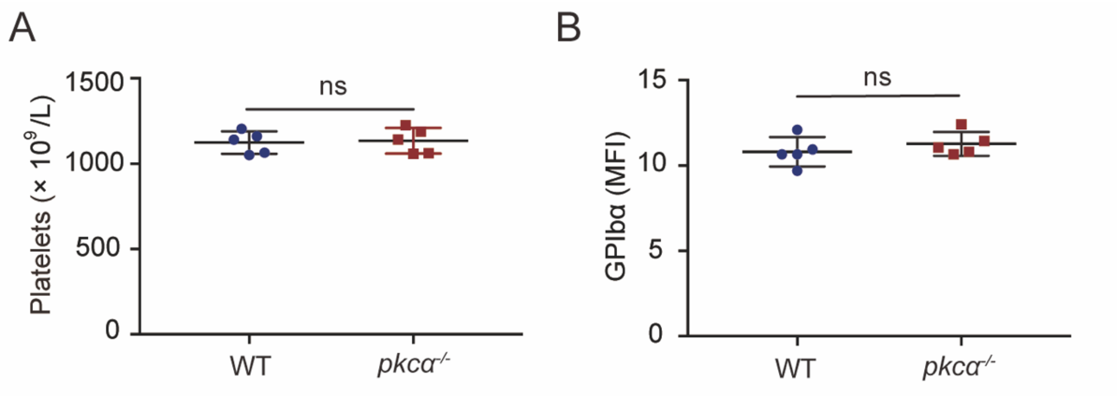

Figure S8. Counts and GPIbα expression of WT and *pkca*-/- mouse platelets

A, Platelet counts in WT and *pkca*-/- mice (n = 5 mice per genotype). **B**, Surface levels of GPIbα on WT and *pkca*-/- platelets analyzed by flow cytometry (n = 5 mice per genotype). ns, not significant.

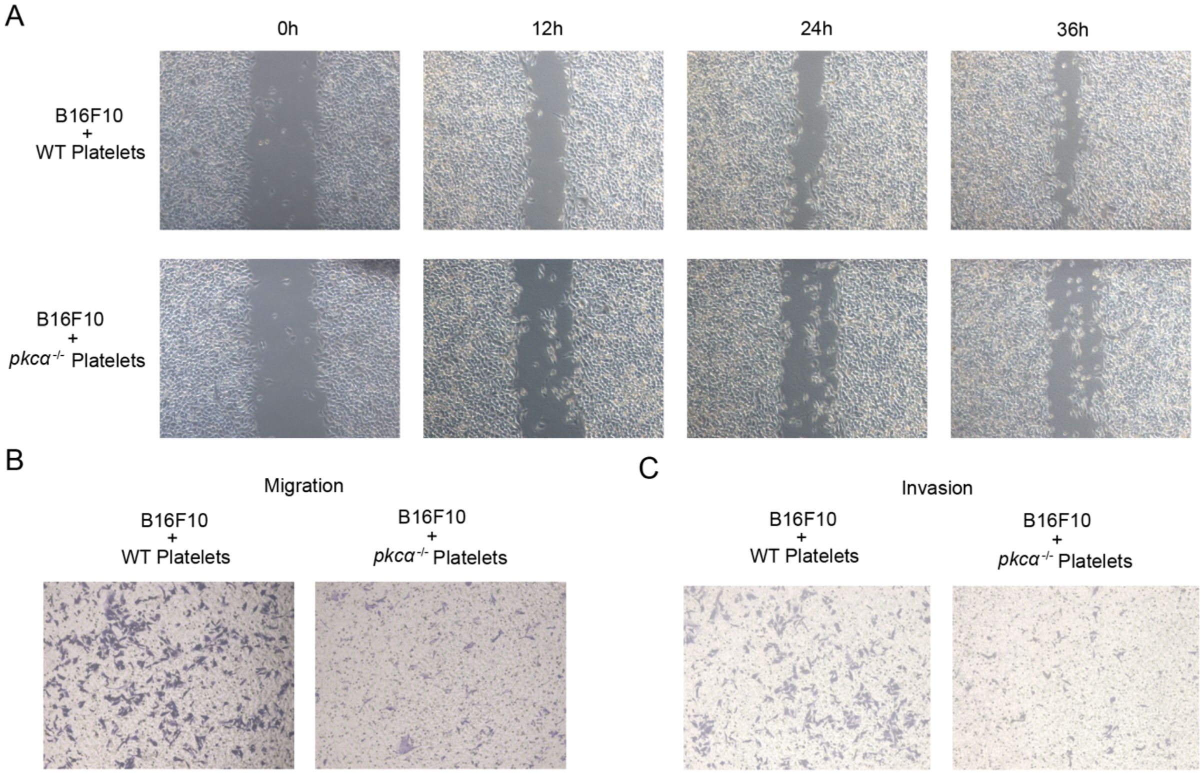

Figure S9. Representative wound-healing and transwell assays images

A, Representative wound-healing images of B16-F10 cells incubated with washed WT and *Pkca*$^{-/-}$ platelets. **B** and **C**, Representative migration (**B**) and invasion (**C**) images of B16-F10 cells incubated with washed WT and 10aa$^{-/-}$ platelets.

Videos' Legends

Video S1 Representative SDIM video of the lung in WT mice 5 minutes after intravenous injection of B16F10 cells.

Video S2. Representative SDIM video of the lung in $10aa^{-/-}$ mice 5 minutes after intravenous injection of B16F10 cells.

Video S3 Representative SDIM video of the lung in WT mice 6 hours after intravenous injection of B16F10 cells.

Video S4. Representative SDIM video of the lung in $10aa^{-/-}$ mice 6 hours after intravenous injection of B16F10 cells.

Video S5. WT mice were intravenously pre-injected with DMSO 10 minutes before the intravenous injection of B16F10 cells. Representative SDIM video of the lung in the mice 6 hours after intravenous injection of B16F10 cells.

Video S6. WT mice were intravenously pre-injected with Gö6983 10 minutes before the intravenous injection of B16F10 cells. Representative SDIM video of the lung in the mice 6 hours after intravenous injection of B16F10 cells.

Video S7. WT mice were intravenously pre-injected with Gö6976 10 minutes before

the intravenous injection of B16F10 cells. Representative SDIM video of the lung in the mice 6 hours after intravenous injection of B16F10 cells.

Video S8. Representative SDIM video of the lung in WT mice 6 hours after intravenous injection of B16F10 cells.

Video S9. Representative SDIM video of the lung in $pkc\alpha^{-/-}$ mice 6 hours after intravenous injection of B16F10 cells.

Original western blots

Figure 3D

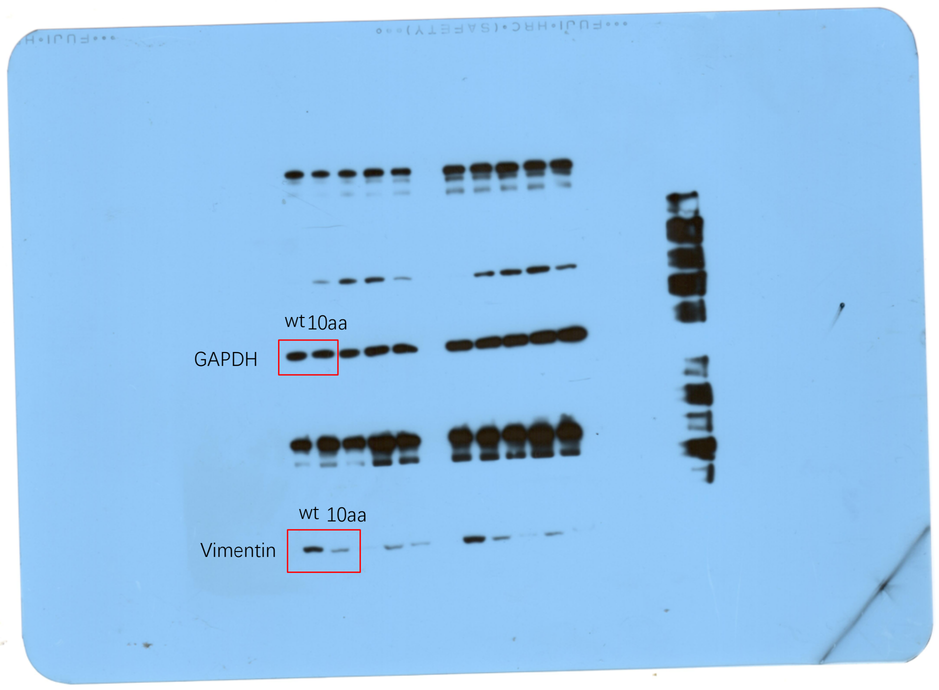

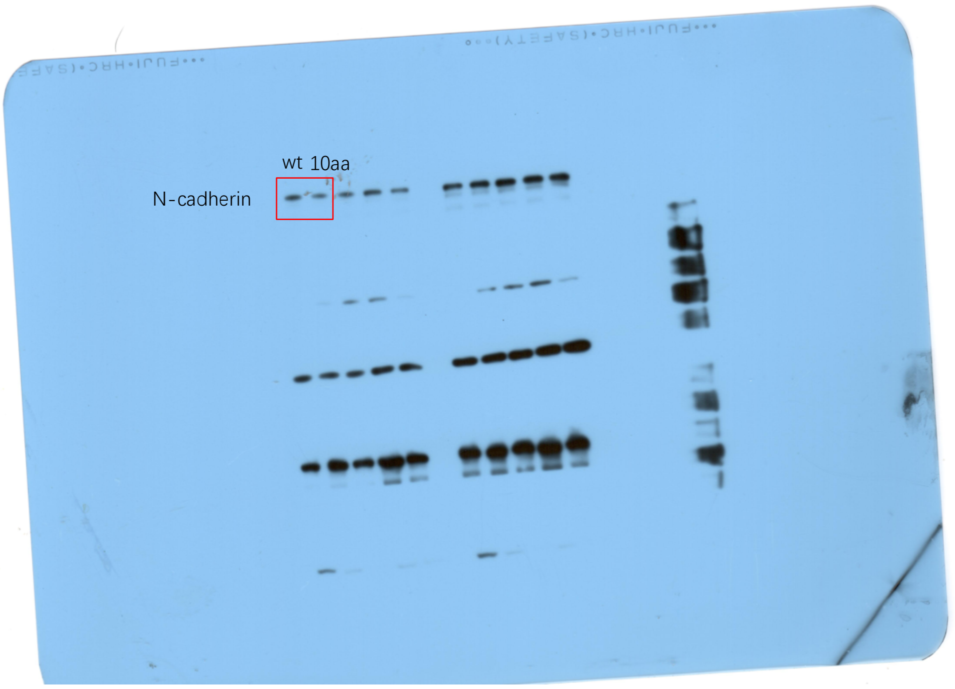

Figure 4A

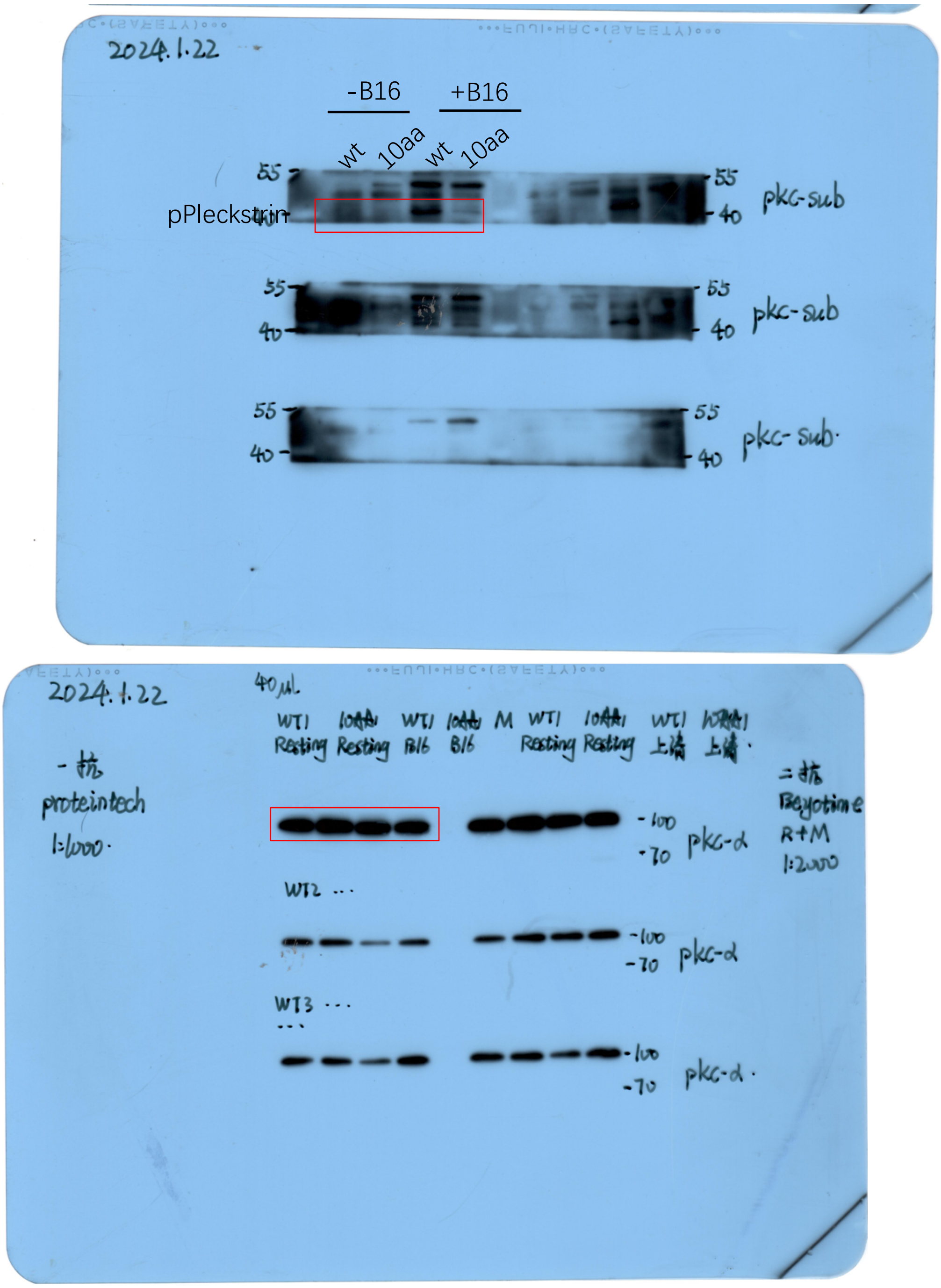
2024.1.22
-B16
+B16
wt
10aa
wt
10aa
pPleckstrin
55
40
pkc-sub
55
40
pkc-sub
55
40
pkc-sub
2024.1.22
40μL
WT1 Resting
10AA1 Resting
WT1 B16
10AA1 B16
M
WT1 Resting
10AA1 Resting
WT1 上清
10AA1 上清
一抗
proteintech
1:1000
二抗
Beyotime
R+M
1:2000
-100
-70
pkc-α
WT2
WT3

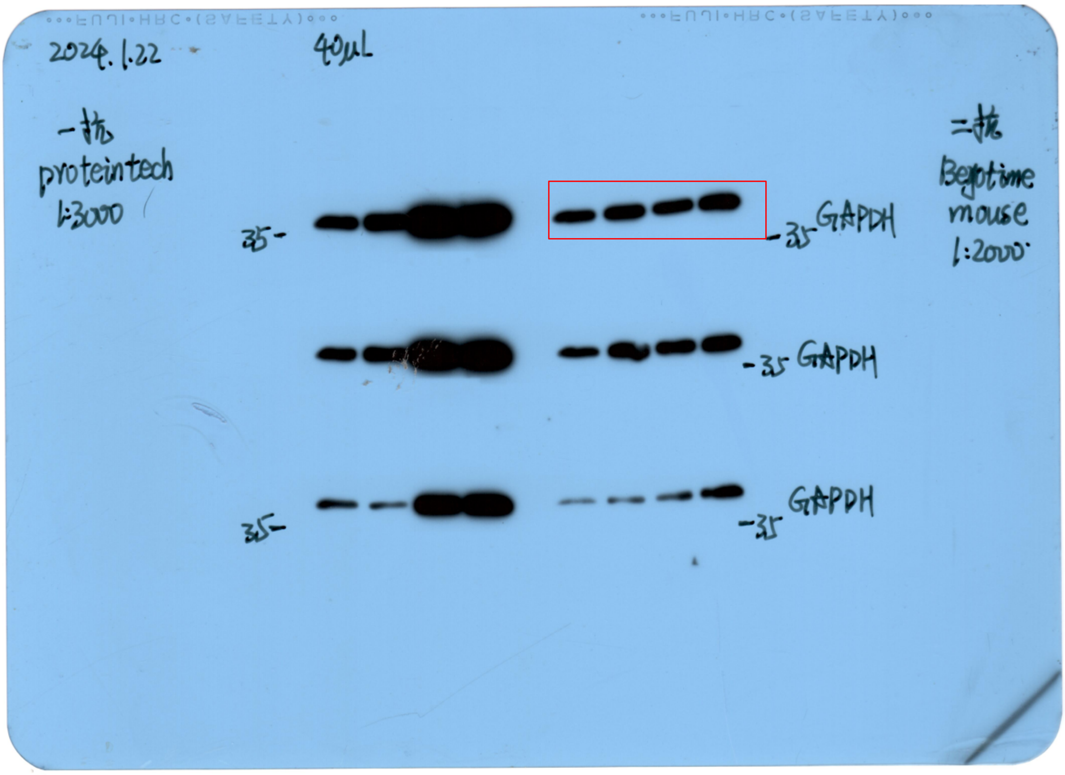
2024.1.22
40μL
一抗
proteintech
1:3000
35
GAPDH
35
GAPDH
35
GAPDH
35
35
二抗
Beyotime
mouse
1:2000

Figure 5A

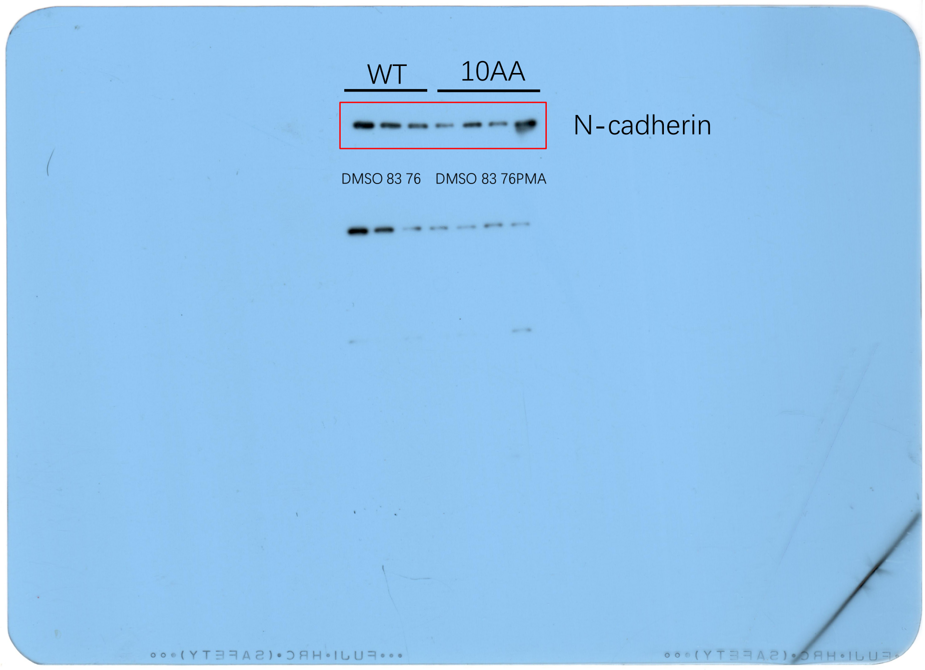
WT
10AA
N-cadherin
DMSO 83 76
DMSO 83 76PMA

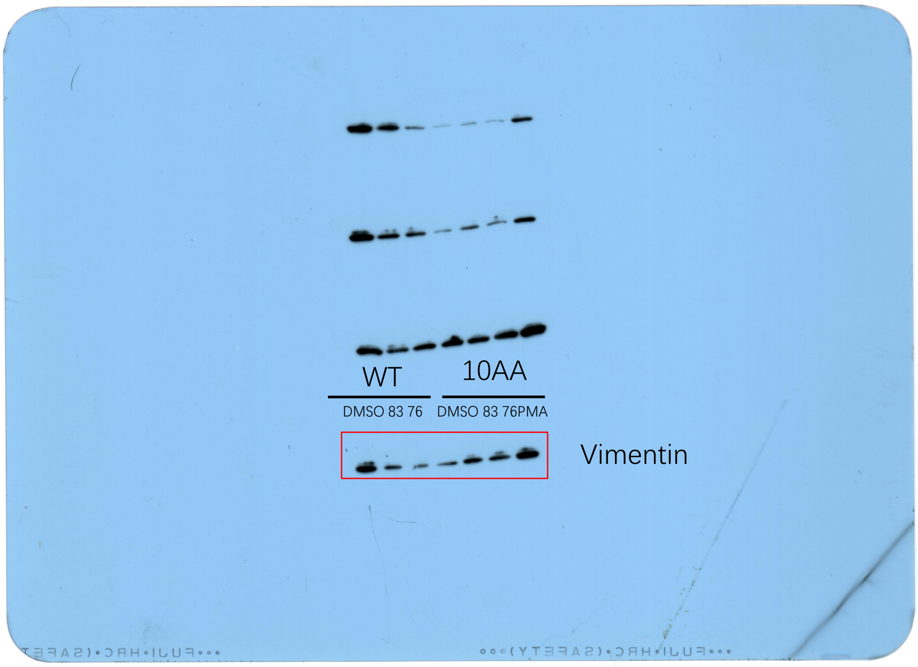
WT
10AA
DMSO 83 76
DMSO 83 76PMA
Vimentin

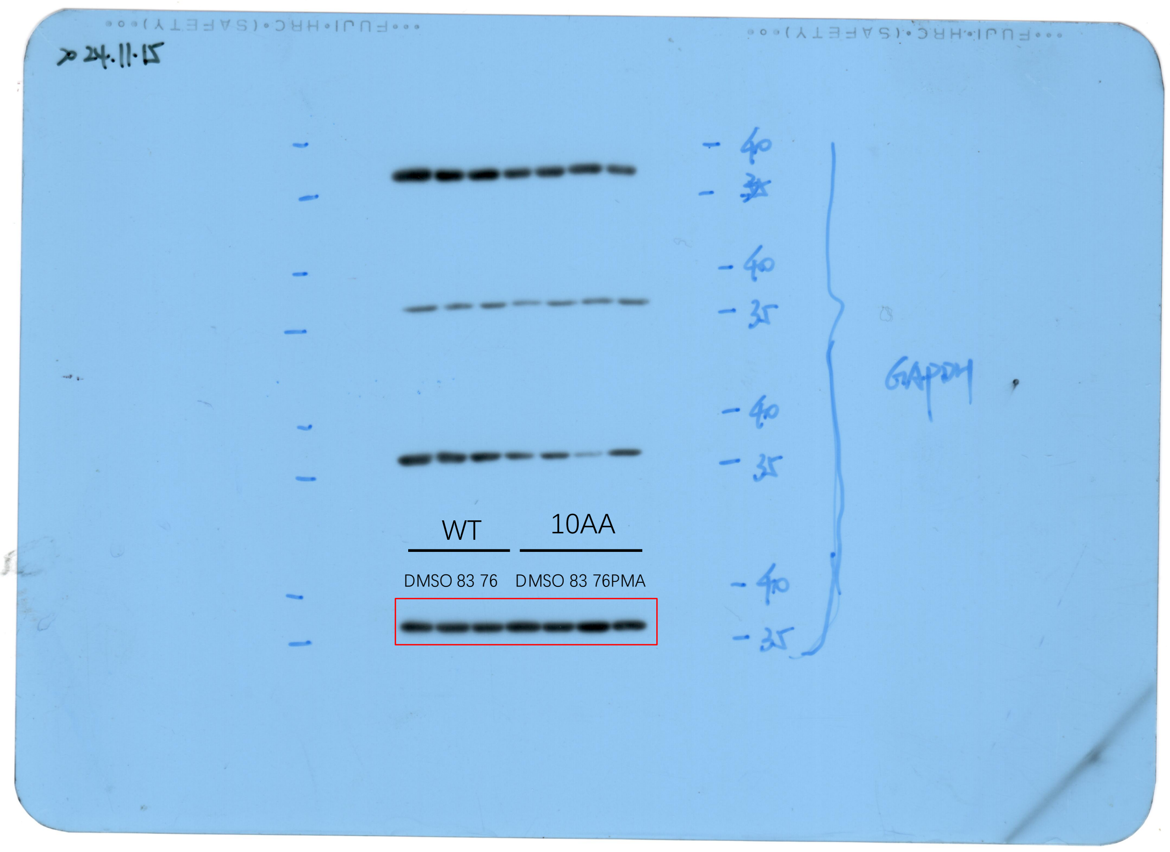

WT
10AA
DMSO 83 76
DMSO 83 76PMA
40
35
GAPDH

Figure 6D

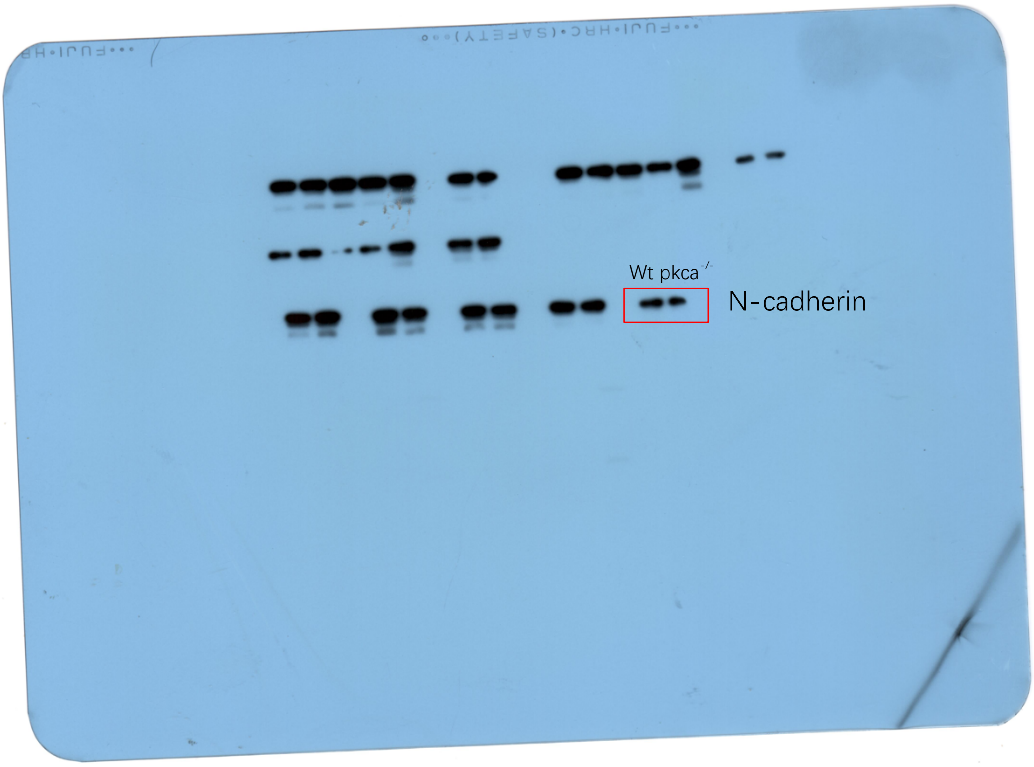

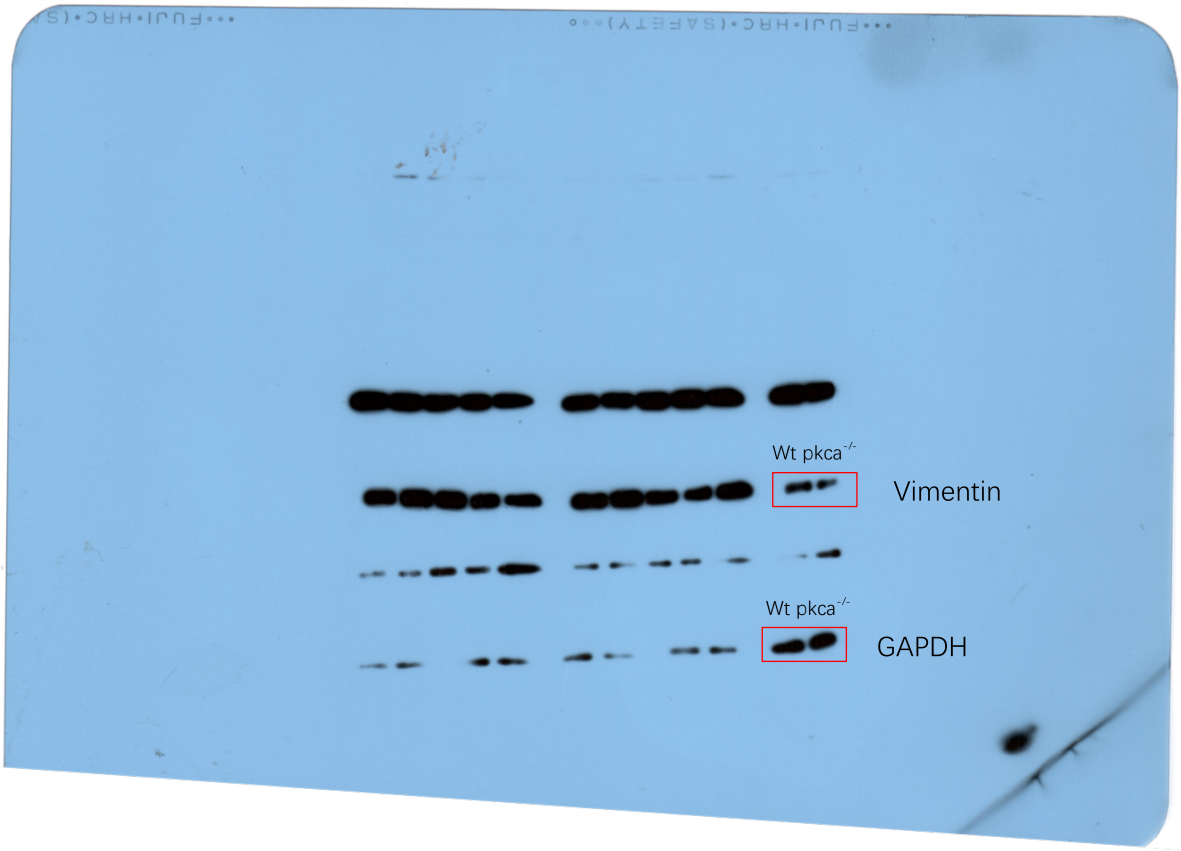

Supplement: Supplementary file 1 — Supporting Information [file MCO2-6-e70217-s001.pdf]
